# Supporting access to healthcare for refugees and migrants in European countries under particular migratory pressure

**DOI:** 10.1186/s12913-019-4353-1

**Published:** 2019-07-23

**Authors:** Antonio Chiarenza, Marie Dauvrin, Valentina Chiesa, Sonia Baatout, Hans Verrept

**Affiliations:** 1 Azienda Unità Sanitaria Locale – IRCCS di Reggio Emilia, Research and Innovation Unit, Via Amendola, 2, 42120 Reggio Emilia, Italy; 2 Université catholique de Louvain, Institute of Health and Society, Clos Chapelle aux Champs 30 B1.30.14, 1200 Brussels, Belgium; 3 London School of Hygiene and Tropical Medicine, Keppel Street, London, WC1E 7HT England; 4 Federal Public Health Service, Food Chain Safety and Environment, Intercultural Mediation and Policy Support Unit, Victor Hortaplein 40 (PB 10), 1060 Brussels, Belgium

**Keywords:** Migrants, Refugees, Asylum seekers, Irregular migrants, Health services accessibility, Europe

## Abstract

**Background:**

In 2015 the increased migratory pressure in Europe posed additional challenges for healthcare providers. The aim of this study was to inform the development of a “Resource Package” to support European Union (EU) member states in improving access to healthcare for refugees, asylum seekers and other migrants.

**Methods:**

A mixed method approach was adopted: i) interviews and focus groups were carried out to gather up-to-date information on the challenges the different healthcare providers were facing related to the refugee crisis; ii) to complement the results of the FGs, a literature review was conducted to collect available evidence on barriers and solutions related to access to healthcare for refugees and migrants.

**Results:**

The different actors providing healthcare for refugees and migrants faced challenges related to the phases of the migration trajectory: arrival, transit and destination. These challenges impacted on the accessibility of healthcare services due to legislative, financial and administrative barriers; lack of interpretation and cultural mediation services; lack of reliable information on the illness and health history of migrant patients; lack of knowledge of entitlements and available services; lack of organisation and coordination between services. These barriers proved particularly problematic for access to specific services: mental health, sexual and reproductive care, child & adolescent care and victim of violence care.

**Conclusions:**

The findings of this study show that solutions that are aimed only at responding to emergencies often lead to fragmented and chaotic interventions, devolving attention from the need to develop structural changes in the EU health systems.

**Electronic supplementary material:**

The online version of this article (10.1186/s12913-019-4353-1) contains supplementary material, which is available to authorized users.

## Background

In 2015 more than a million refugees and migrants crossed the Mediterranean sea to reach Europe [1]. Although the number of those seeking international protection was lower than that of ‘routine’ migrants for purposes such as work, family and study [2], the increased migratory pressure in Europe posed additional challenges for the different actors providing care for these vulnerable groups. The migratory influx generated the presence of different types of migrants who can be categorised according to the phase of the migration trajectory they were in and the type of legal status they were attributed at that particular point. These phases can be divided into arrival, transit and destination, although they are not clear-cut and offer a number of grey zones both for migrants and countries.

As unauthorised entrants, newly arrived migrants were in principle irregular migrants. As soon as they applied for asylum, their presence in the country became legal, but if they moved on to other countries, they returned to irregular status. Refugees were in theory granted protection from formal registration of their application for asylum in the first receiving country. In practice, however, they frequently found themselves in a situation where they had no effective healthcare coverage, awaiting an often long overdue response to their application for refugee status, at times at the stage of an appeal, or even refusal. The number of negative decisions following the peak in applications in the second half of 2015 was 449,920, 39% of the 1.148,680 decisions made in 2016 [3]. Those who had been refused protected status but had not been deported remained irregular migrants.

EU countries have been affected differently, depending on whether they were arrival, transit or destination targets. However, there were similarities in that refugees and migrants persistently faced barriers to access adequate health services. Certain barriers had already been identified, these included restrictive regulations to access healthcare based on legal status, linguistic and cultural barriers, lack of information regarding where and how obtain care, economic barriers, and lack of cultural competence among health providers [4]. Nevertheless, as reported in the Migrant Integration Policy Index (MIPEX) Health strand in 2015, although the level of implementation of EU national policies addressing these barriers was particularly low in Eastern European countries, the lack of policies aiming at ensuring the right to healthcare still regarded the majority of EU countries [5].

The aim of this study was to inform the development of a ‘Resource Package’ (RP) to support health authorities, both at national and local level, improving access to appropriate health care services for refugees and migrants. The study was part of the EU project ‘Supporting health coordination, assessments, planning, access to healthcare and capacity building in member states under particular migratory pressure’ (SH-CAPAC).^1^ Specific objectives were: i) to collect up-to-date information on the challenges facing the different healthcare providers, governmental and non-governmental organisations (NGO) as well as international and civil society organisations related to the refugee crisis; ii) to investigate how these challenges impacted on the accessibility of healthcare services; iii) to identify the measures and tools healthcare providers put in place to improve accessibility; iv) to complement this information with the available evidence on the barriers and solutions related to access to healthcare for migrants.

## Methods

A mixed method approach was adopted: firstly, a series of interviews and focus groups (FG) were carried out to gather up-to-date information on the challenges that healthcare providers were facing in providing healthcare for refugees and migrants; secondly a systematic review of the literature was conducted, to collect, summarize and critically appraise the available evidence on barriers and solutions related to access to healthcare services for these vulnerable groups.

### Focus groups and interviews

Between February and March 2016 ten FGs and twenty individual semi-structured interviews were carried out in ten EU countries, characterised as being primarily arrival, transit or destination countries. FGs/interviews were conducted in Greece, Italy, Spain (arrival countries); Slovenia, Hungary (transit countries); Austria, Belgium, Denmark, The Netherlands, United Kingdom (destination countries). Purposeful sampling was utilised to identify key persons to conduct the FGs/interviews. Key persons were expert researchers who might be expected to have a specific knowledge of the migrants’ situation in their countries. Specifically, they were members of European research networks familiar to the authors.^2^ Each expert researcher was provided with a FG/interview guide (Additional file 1) and instructions on how to obtain informed consent from participants for the audio-recorded FG/interview. Participants were healthcare providers and managers working in reception centres, as well as in mainstream health services. In total 128 healthcare providers participated in the FGs and interviews (Table 1).

**Table 1:** Occupation and organisation of health providers participating in FGs and interviews, by country (*n* = 128)

	Interviews (*n* = 20)			Focus groups (*n* = 10)							Total n (%)
	Austria (*n* = 6)	Netherlands (*n* = 4)	UK (*n* = 10)	Belgium (*n* = 2)	Denmark (*n* = 1)	Greece (*n* = 2)	Hungary (*n* = 1)	Italy (*n* = 1)	Slovenia (*n* = 1)	Spain (*n* = 2)	
Occupation											
Medical doctor	2	6	3	2	3	4	3	1	4	3	31 (24)
Service manager	1	–	–	4	4	1	4	2	1	1	18 (14)
Nurse	2	–	4	2	–	6	1	3	2	2	22 (17)
Psychologist	–	–	–	2	1	2	–	2	–	7	14 (11)
Social worker	–	–	–	3	–	1	2	2	–	4	12 (9)
Intercultural mediator	–	–	–	6	–	–	–	1	–	2	9 (7)
Activist	–	–	3	1	1	1	1	–	1	–	8 (6)
Administrative staff	–	–	–	–	1	3	–	3	–	–	7 (5.5)
Other	1	–	–	1	–	–	1	–	–	4	7 (5.5)
Organisation											
National Health System	4	2	3	10	5	11	1	9	–	5	50 (39)
NGO	–	2	4	2	2	2	1	3	–	3	19 (15)
Reception centre	–	–	–	2	–	–	3	–	7	4	16 (12.5)
Government agency	–	2	–	2	2	1	–	1	–	4	12 (9.5)
Caritas & charity	2	–	–	2	–	–	4	1	–	2	11 (8.5)
Doctors of the World	–	–	–	3	–	2	–	–	–	2	7 (5.5)
University	–	–	3	–	–	2	–	–	–	1	6 (5)
Professional association	–	–	–	–	–	–	3	–	–	1	4 (3)
Red Cross	–	–	–	–	1	–	–	–	1	1	3 (2)

FG/interviews were conducted in the language of the 10 countries involved. Descriptive and analytical notes were taken immediately after the interviews and the FGs. Each expert researcher produced a brief report in English, summarising the main findings grouped into three thematic areas: ‘challenges for healthcare providers and managers’; ‘solutions to address the challenges’; and ‘development and dissemination of a RP’. National summaries were then comparatively analysed by the two researchers responsible for the study. These excerpts were coded manually, categorised, and analysed, applying the six-phase approach to thematic analysis [6].

### Systematic literature review

To add evidence to the findings of the interviews and FGs, a systematic review (SR) was conducted in July 2016. The research question was: *“What are the current barriers and solutions related to access to health services for asylum seekers and refugees in OCDE countries?”.* The search strategy initially designed for the Medline database based on the PICO method was then adapted to other databases: CINHAL, Embase, Scopus, the Cochrane Database and CAIRN. The SR followed the PRISMA guidelines and the methodology was defined a priori. Grey literature was examined manually: key websites were searched for additional resources, together with the abstract books of the last 3 European public health conferences (Granada 2014, Milano 2015, Oslo 2016).

### Inclusion and exclusion criteria

Studies were included if they (i) were published between January 2008 and July 2016; (ii) were written in English, French, Italian, Spanish and Dutch; (iii) had the geographical focus of one or more of the EU or OECD countries; (iv) reported, original qualitative, quantitative or mixed data; (v) focused on or specifically included refugees and migrants as study participants; (vi) reported data on barriers related to access and/or on interventions aiming at decreasing access barriers for migrants and/or refugees; (vii) regarding grey literature, if they were reports from NGOs and official institutions (e.g. WHO, EU or OECD). Studies were excluded if they (i) did not report original data, such as letter to the editor, comments, book reviews or editorials; (ii) focused only on labour migrants, ethnic minorities, internally displaced populations; (iii) focused only on epidemiological aspects; (iv) focused on integration aspects without any reference to healthcare accessibility; (v) presented research methods, instrument development, theoretical models without application.

Data were extracted by two authors and supervised by a third author using a standardised data extraction spread sheet. References were stored in an Endnote library. Standardized review forms were used to retrieve the following data: 1) general information on the study; 2) data on the study population; 3) health care provision; 4) health care settings; 5) barriers preventing access to health care services; and 6) solutions to improve access to health care. Identified studies were independently reviewed for eligibility by two authors in a two-step process; a first screen was performed based on title and abstract and full texts were retrieved for the second screen. In case of disagreement in the selection process, a third reviewer was consulted. Studies were only included if all reviewers agreed.

## Results

Findings of both the interviews/FGs and the SR are presented in the following sections: (1) challenges related to specific phases of the migration trajectory (2) barriers and solutions related to accessing healthcare services in general: legislative, financial and administrative aspects; linguistic and cultural issues; information for healthcare providers; information for refugees and migrants; organisation and quality of services; lack of coordination between care providers; (3) barriers and solutions related to accessing four specific healthcare services: mental health, sexual and reproductive care, child care and victim of violence care; (4) development and dissemination of a resource package.

**Fig. 1:**
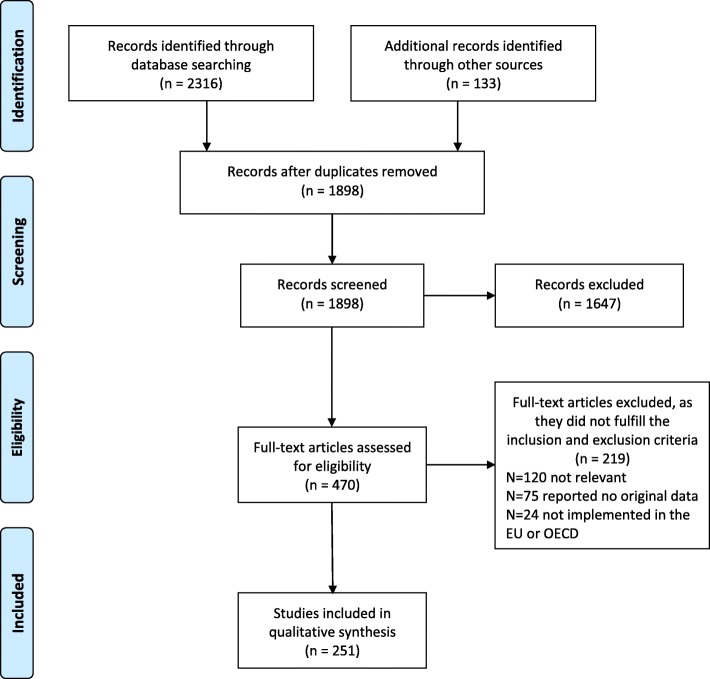
PRISMA flow diagram of papers selected

Figure 1 presents the flow process of the literature review: after reviewing 2316 references, 251 studies were included in the final database for analysis. Table 2 describes the characteristics of the included studies. The majority of the studies were conducted at the destination phase (*n* = 201); only 3 concerned the transit phase. The most cited setting was the health-care system level (*n* = 167); only 3 were found for accident and emergency services. The target group mostly addressed was refugees (*n* = 136); 55 studies addressed health providers; 88 both refugees and health providers and 22 policymakers. No relevant difference between target groups was observed with regard to general barriers concerning access to healthcare services nor in access to specific healthcare services. A complete list of these studies is described in Additional file 2.

**Table 2:** Characteristics of the included studies on barriers and solutions to access healthcare services by type of service ^a;b^

	Type of service					Total number of studies (n)
	Healthcare services in general (*n* = 131) ^[1–131]^	Specific healthcare services (n = 135)				
		Mental health (*n* = 50) ^[132–181]^	Child care (*n* = 30) ^[182–211]^	Victim of violence care (*n* = 10) ^[212–221]^	Sexual and reproductive care (*n* = 45) ^[222–266]^	
Healthcare setting						
• Health care system	81	33	18	6	29	167
• Primary care service/GP	30	9	5	3	8	55
• Specialised services	5	7	6	2	6	26
• Reception centres	11	2	2	–	2	17
• Hospital services	3	4	4	0	4	15
• Accident & emergency departments	1	1	–	–	1	3
• Other settings (school, community …)	6	5	6	–	5	22
Migration phases						
• Arrival phase	9	2	2	–	–	13
• Transit phase	3	–	–	–	–	3
• Destination phase	94	39	21	6	41	201
• All	15	2	6	–	1	24
• Not specified	13	7	2	4	3	29
Participants included in the study						
• Refugees/asylum seekers	63	28	15	2	28	136
• Health providers	22	13	8	4	8	55
• Refugees/Asylum seekers and health providers	48	11	12	3	14	88
• Policymakers	11	3	5	3	–	22

^a^Please note that a same study could focus on more than one type of health-care setting, migrations phase, participant and on different specific health care services

^b^Please refer to Additional file 2 for the references included in the Systematic Review

Table 3 presents the key results of the SR classified according to the themes identified from FGs and interviews results. In total 451 barriers and 335 solutions were identified across the 251 analysed studies. The majority of the barriers and solutions concerned the access to the healthcare system in general (*n* = 131), with a focus on linguistic and cultural issues (barriers = 65, solutions = 44). Regarding specific health services, mental health services were mostly addressed (barriers *n* = 81, solutions *n* = 64). Victims of violence were poorly addressed (barriers n = 13, solutions *n* = 12). The SR chose to focus on mental health, sexual and reproductive care, child care and victim of violence care as they were identified as priority areas by FGs/interviews findings. A complete list of the included studies divided by barriers and solutions is described in Additional file 3.

**Table 3:** Distribution of barriers and solutions on access to health care in the retrieved studies (*n* = 251) ^a^

	Healthcare services in general *n* = 131 ^[1–131]^		Mental health *n* = 50 ^[132–181]^		Child care *n* = 30 ^[182–211]^		Victim of violence care *n* = 10 ^[212–221]^		Sexual and reproductive care *n* = 45 ^[222–266]^		Total	
	Barriers n (%)	Solutions n (%)	Barriers n (%)	Solutions n (%)	Barriers n (%)	Solutions n (%)	Barriers n (%)	Solutions n (%)	Barriers n (%)	Solutions n (%)	Barriers n (%)	Solutions n (%)
Categories												
Legislative and administrative aspects	44 (19)	25 (13)	8 (10)	6 (9)	15 (31)	7 (14)	3 (23)	3 (25)	11 (14)	2 (9)	81 (18)	43 (13)
Linguistic and cultural issues	65 (30)	44 (23,5)	32 (39)	15 (23)	14 (30)	12 (24)	5 (38)	2 (16,6)	27 (35)	9 (41)	143 (32)	82 (24,5)
Information for health providers	19 (8)	17 (9)	–	2 (3)	–	2 (4)	1 (8)	1 (8,4)	4 (5)	–	24 (5)	22 (6,5)
Information for refugees and migrants	36 (15)	27 (14)	13 (16)	8 (12,5)	7 (15)	8 (16)	1 (8)	–	14 (18)	3 (13,5)	71 (16)	46 (14)
Organisation and quality of services	46 (20)	45 (24)	25 (31)	25 (39)	10 (20)	13 (26)	3 (23)	6 (50)	20 (26)	7 (32)	104 (23)	96 (28)
Coordination between care providers	21 (9)	29 (15,5)	3 (4)	8 (12,5)	2 (4)	8 (16)	–	–	2 (2)	1 (4,5)	28 (6)	46 (14)
Total	231 (100)	187 (100)	81 (100)	64 (100)	48 (100)	50 (100)	13 (100)	12 (100)	78 (100)	22 (100)	451 (100)	335 (100)

^a^Please note that a same study could focus on more than one barrier or solution

### Challenges related to specific phases of the migration trajectory

#### Arrival

Participants of FGs/interviews from arrival countries reported that refugees arrived in large numbers and usually stayed for relatively short periods of time, days or even hours. The numbers frequently overcame the capacity of existing health and support services, creating a humanitarian crisis situation. It was reported that primary health care interventions were provided on site during the arrival phase. Red Cross, Médecins du Monde (MdM), Médecins sans Frontières (MSF) along with other NGO’s were the main care providers. Here, participants described a situation characterised by a lack of coordination between the many different organisations involved in healthcare provision. At the time that FGs were conducted this situation tended to be more problematic in places affected by a huge influx of migrants as in the case of Lesvos, Greece, because of the high number of patients to be seen by healthcare professionals. As a result, migrants who needed psychosocial support, or treatment for chronic diseases often finished in hospital emergency departments. Participants reported that because of limited time in this phase, emergency interventions were provided with absolutely no integration of care, therefore it was very difficult to have a complete clinical picture of the patient. As a consequence, often chronic diseases or psychological disorders and migrants’ personal plans were not taken into account.

#### Transit

FG/interview participants from transit countries reported that the main concern of refugees and migrants was to continue their journey to their destination country. In this phase NGOs continued to be the main onsite providers of care. If the health problem was considered serious, refugees were taken to hospital, but they often did not complete treatment as they wanted to continue to Northern Europe. As a result, it was reported, that treatment of chronic diseases was often inadequate. Lack of personal medical files was highlighted in many FG discussions, as in every new health care setting all relevant medical data needed to be collected once again. In this situation there was little opportunity for implementing prevention and promotion programmes, since the focus was mainly on acute health issues and communicable diseases. Time was reported to be one of the main challenges when it came to refugees in transit. For example, pregnant women were exhorted to take specific tests to assess their own and their baby’s health, however, taking into account the waiting times and procedures of EU health systems, access to these services proved to be very complicated. The same challenge was reported for urgent psychological assistance and mental healthcare.

#### Destination

Health problems began to become a concern when refugees and migrants reached destination but the ongoing healthcare required, needs to be integrated into the mainstream health care system. Participants reported that this implied that certain services that had been provided earlier free of charge before recognition of refugee status, were later subject to payment by migrant patients. It was reported that this was the case for outpatient mental health services in Belgium and led to financial barriers. Interviewees argued that at destination, refugees lost much of the support they might have received during the previous phases. Now, refugees and migrants would be expected to access and use mainstream health and social services unassisted. This proved, in many cases, challenging because of linguistic and cultural barriers as well as the migrant’s low health literacy and lack of knowledge of the bureaucratic and complex healthcare system. The impacts of these barriers, it was argued, were intensified by the limited cultural competence of many health providers.

### Barriers and solutions related to accessing healthcare services in general

#### Legislative, financial and administrative aspects

Overall, findings of both the FGs/interviews and the SR identified legal status as the most important aspect directly affecting health and social services access for refugees and migrants. [7–10]. As emphasized in one Health Evidence Network (HEN) report, although the formal introduction of application for asylum ensures migrants’ right to access health services once registered in the receiving country, in practice, administrative barriers and the lengthy procedures necessary to obtain entitlement impede asylum seekers from receiving full healthcare coverage [7]. FG/interview participants reported that different procedures had to be followed depending on the migratory status of the asylum seeker. One study outlined that two groups of migrants are particularly at risk: those between legal positions, in the transition from asylum seekers to refugees; and those who failed to seek asylum and became irregular migrants [11]. These, it was argued, may be left at an impasse with no right to treatment, no means to pay, and a sole entitlement to emergency healthcare. Another aspect highlighted by FGs/interviews was that care providers were often insufficiently informed of relevant legislation. Little familiarity was reported among care providers of the different legal statutes of asylum seekers, refugees and irregular migrants and what their entitlements really were. As a consequence, migrant patients were often unable to exercise their rights to care. Finally, several studies emphasized that irregular migrants and those who are not entitled to apply for asylum do not seek health care to avoid the risk of being deported [7–10].

As suggested by the United Nations High Commissioner for Refugees (UNHCR), the most effective way to improve access to services is to remove legal constraints and any inequitable practices that hinder access to healthcare [12]. The UNHCR also pointed out that solutions should aim, on the one hand at ensuring unconditioned healthcare coverage for all migrant groups through inclusive national legislation and, on the other hand, at reducing the bureaucracy and lengthy time to process documentation. To this purpose, health managers and decision makers need to analyse the relevant laws and regulations in their country, and identify solutions to financial and administrative barriers, as in the cases reported by two FGs: in Belgium, in one hospital, they introduced “vouchers for free consultations for un-insured patients”; in Italy, in the local health authority of Reggio Emilia they created a “dedicated healthcare service for irregular migrants”. Finally, findings from FGs/interviews stressed the need to make healthcare providers more aware of the legislation affecting asylum seekers, refugees and irregular migrants, and sensitive to the fact that access to healthcare should not entail any form of reporting to the authorities.

#### Linguistic & cultural issues

Lack of interpreting services was identified by many studies as an important barrier to effective healthcare for refugees and migrants [13–17]. FG/interview participants systematically reported linguistic barriers as one of the main challenges they faced providing care for migrants and refugees. When available, interpreting services were often carried out by NGO members, volunteers or other migrants, with no specific professional qualifications and as a consequence, care was often provided on the basis of poor communication. The inability to solve linguistic barriers made it very difficult to deal with the cultural barriers and this further hindered the process of care provision.

In accordance with most studies examined [15, 16], FGs/interviews reported that solutions to overcome linguistic and cultural barriers would be the systematic accessibility of interpreters and/or intercultural mediators at all levels of care. Different options on how the services of professional interpreters and cultural mediators could be obtained depend on the characteristics of the health service and its language needs and are described in the literature [16]. Furthermore, a Swiss study pointed out that clear organisational policies setting out how interpretation and intercultural mediation services are provided should be defined and be part of the overall development of a culturally competent healthcare system [17].

#### Information for healthcare providers

FGs/interviewees reported that refugees and migrants frequently arrived in receiving countries without any medical records. In particular healthcare providers emphasized that the lack of children’s health records was a significant problem and, because of language barriers, information on the vaccination status of children could not be acquired from parents. The lack of reliable health information for healthcare providers was also highlighted in a number of studies [10, 18]. Furthermore, FG discussions reported that since there was no adequate system for the exchange of health data across EU countries, traceability of patients moving from one country to another was often impossible. They outlined that even within one country, moving from one place to another, or from one type of healthcare service to another may have entailed the loss of relevant information on the health status and medical history of the patient.

As a solution, FG/interview participants envisaged the establishment of an European system to exchange health information that would allow migrants to access and share their medical data wherever they are thus ensuring better quality and continuity of care. This issue was addressed by a study conducted by the International Organization for Migration (IOM) describing the development and pilot testing of an electronic Personal Health Record (e-PHR) with the aim to ensure that migrant health assessment records were available along the migration journey [18]. Another study proposed the introduction of patient-held records [11], in order to have medical information travelling with the patients. However, the results of a Dutch study [19] shows that the use of patient-held records was low, because neither the undocumented women nor general practitioners involved in the study, considered it to be a solution.

#### Information for refugees and migrants

The lack of knowledge of entitlements and available services on the part of refugees and migrants were perceived by FGs/interviewees as the major obstacles to accessing healthcare and using services appropriately. In particular, they reported that refugees and migrants showed a poor understanding of primary healthcare and the appointment system. The primary issues identified in many studies were the absence of health service information for migrants upon arrival in the country and difficulties in navigating the health system [4, 9, 20]. Specific challenges included difficulties in accessing specialist services, understanding explanations of treatments and participation in health promotion and disease prevention programmes [21, 22].

A range of strategies for the provision of effective information for migrants and refugees were outlined in selected studies [22, 23]. These strategies may include provision of language-appropriate written material, the use of intercultural mediators and/or community health educators to facilitate health promotion and education programmes. A good example reported in one FG in Spain involved a strategy to inform migrants about their rights. This had been implemented in the region of Andalusia and jointly developed by the NGO ‘Plataforma Somos Migrantes’^3^ and the Department of public health.

#### Organisation and quality of services

Limited availability of services was reported in FG discussions as supplementary healthcare services were provided in few countries. Poor healthcare management of the refugee influx, participants argued, often led to overcrowding of hospitals and longer waiting times. Unequal distribution of facilities, lack of transportation, increased dependence on accident and emergency services were often described in selected studies, emphasising the need to improve access to primary care [7, 10, 13, 24]. Interviewees/FGs referred that some private hospitals did not want to treat refugees and asylum seekers because of the risk of non-payment and administrative burdens. Furthermore, access to specialized care was sometimes hampered by the fact that care providers are allowed to set their own fees since the State (e.g.: Belgium) only reimburses amounts fixed by the national health insurance system.

Several studies outlined that improving access to and quality of healthcare for refugees and migrants is a primary responsibility of organisations and health systems [24–27]. One study reported that an effective solution would be the adoption of a ‘whole organisational approach’ able to implement a comprehensive process of change [26]. Within this framework, healthcare organisations need to develop specific programmes that address priorities for refugees and migrants, adapt processes and services and effectively train staff at all levels in order to deliver quality healthcare services in a coordinated and equitable fashion. Other solutions reported by two other studies are the co-location of different health services, the promotion of out-reach services and free transportation, and the implementation of drop-in primary healthcare units based in hospitals [24, 27].

#### Lack of coordination between healthcare providers

Most of the FG participants argued that the presence of different NGO’s and groups of volunteers, combined with a lack of organization, contributed to chaotic and inadequate collaboration between the different healthcare providers. It was noted that uncoordinated interventions by many different care providers, often failed to meet healthcare needs, merely leading instead to overlapping and duplication. One study found that the complex relationships between organisations can lead to confusion for refugees and migrants preventing them from finding their way through such complicated systems [23]. Furthermore, a report from IOM emphasized that the lack of coordination not only creates confusion in the division of tasks but also produces a misuse of human and economic resources [28].

Developing health coordination mechanisms to bring together all participating stakeholders involved in the health response to the influx of refugees emerged as crucial from all FG discussions. A report from UNHCR highlighted that a partnership with a wide range of actors, especially government (Ministries of Health, Interiors and Foreign Affairs), United Nations and international organisations (IOM, WHO), NGOs (Red Cross, MdM; MSF), and civil society organisations is necessary to ensure the availability of quality public health services for refugees and migrants [12]. However, most participants of FGs/interviews stressed the urgent need to improve coordination between these different partners. One study emphasized that this coordination should begin at the planning stages of service delivery [29]. In particular, shared and horizontal protocols involving multiple sectors and levels would ensure coordination and quality of care, as demonstrated by the WHO ‘contingency plan’ implemented in Sicily [30].

### Barriers and solutions related to accessing specific healthcare services

#### Mental health service

Interviews/FG participants working in arrival camps reported that they met a great number of refugees in need of psychosocial assistance and support. They explained that this was due to the situation and traumatic experiences migrants often encounter before and during their journey. Furthermore, participants highlighted that repressive police actions, extended asylum procedures, unexpected displacements, threat of deportation might lead to psychological disorders. In spite of all that, participants reported, there was insufficient psychological support to help refugees with traumatic experiences and there were difficulties in accessing specialist therapies. Some participants outlined that specialist care was covered, but only in limited range to vulnerable persons with special needs (e.g.: victims of trafficking, torture or sexual violence) and on condition of approval by a special commission.

Many respondents pointed out that to overcome these challenges, it would be essential to improve the presence of well-trained professionals especially at arrival/transit phases. Furthermore, they stressed the importance of adopting alternative approaches to traditional mental health services. As highlighted in the literature, adopting a narrative approach that is informal yet respectful, keeping the person at the centre without being judgemental, proves successful in overcoming barriers to access psychological advice or treatment. [31], as do strategies to remove services from the stigmatised context of mental health settings to places that are more acceptable for refugees and migrants [32]. Finally, establishing forms of collaboration between mental health services, schools and refugee support organisations, as well as improving information on available services for both migrants and health care professionals, was reported by a British study to be an effective strategy to overcome the many barriers to access [33].

#### Sexual and reproductive care

A MdM report argued that national regulations heavily affects access to sexual and reproductive health (SRH) services [34]. Furthermore, a HEN review [35] identified affordability as the major barrier to access maternal healthcare. This was confirmed by FG/interview findings highlighting that pregnant women were only registered in the health service system at a late stage in their pregnancy, probably because in some countries antenatal care was charged. Scarce knowledge of contraception, sexual health or sexually transmitted diseases, as well as lack of recognition of postnatal depression were among the major issues reported in the HEN review [35]. In addition, both SR and FG results outlined that barriers to accessing SRH are determined, on the one hand, by the lack of information and familiarity with the health system by migrant women, and on the other hand, by the lack of knowledge on legal issues by healthcare workers, who ignore the legal framework and the respective entitlements [36].

The first most important solution highlighted in the literature [35] and advocated by healthcare providers was the implementation of inclusive national policies allowing the provision of full healthcare coverage for all migrant pregnant women and for their children regardless of legal status. Secondly, the importance of ensuring precise information on services available for migrant women during pregnancy, childbirth, and the postpartum period and their right to access them was stressed. Other strategies such as counselling interventions and family planning are presented by the HEN review as efficient methods to enhance the health of migrant women and avoid unwanted pregnancies [35]. Finally, an Irish study [37] reported that the provision of community-based interventions in primary care settings involving health professionals, midwives and community health educators would improve not only accessibility to maternal care but also continuity of care for migrant women.

#### Child care

Children and adolescents are among the most vulnerable groups in the migrant population. As reported by one study, unaccompanied or separated refugee minors are at major risk. They experience not only the difficult condition of living without their parents but often the traumatic consequences of being subject to violence, abuse and exploitation [38]. Furthermore, another study [39] emphasised that children who are separated from their families and have no residence permit are likely to become undocumented migrants, thus, as outlined in a PICUM report [40], risk the consequent bureaucratic barriers to access appropriate health care [41]. On top of all that, one study pointed out, migrant children risk facing legal barriers linked to age determination, as long as age influences access to care if over 18 years.

FGs/interviewees also stressed that bone age assessment was a source of anxiety and unreliable, thus it would be preferable to identify more effective guidelines for age assessment and family tracing. Health promotion and prevention strategies, migrant communities and NGOs engagement, as well as improved information and training of health professionals on refugee children and adolescents’ health-related issues were highlighted by one study [42], as well as in the FGs and interviews, as effective strategies to address these barriers.

#### Victims of violence care

Many studies stated that refugees and migrants suffer from persecution and torture in their country of origin and a great number of women, adolescents and children experience physical and/or sexual violence along the migratory route [35, 43, 44]. One study argued that these traumatic experiences continue to have a psychological impact on migrants’ lives in their destination country and are key factors preventing access to appropriate health and social care [43]. Furthermore, another study found that sexual and gender-based violence (SGBV) is also a significant problem among refugee populations and has important consequences for their physical and mental health, such as injuries, infections, unwanted pregnancy, infertility and a wide range of emotional, cognitive and behavioural disorders [35]. Despite the evident need to ensure access to appropriate services for the victims of torture and violence, the implementation of the Istanbul protocol to prove an individual suffered torture is a challenge due to the high cost of the expert report, as was reported by FGs and interviewees.

One study [45] reported that stigma, discrimination and fear of being excluded by family and community members often impede victims of violence from seeking the care they actually need. It is not surprising that the lack of trust between health providers and violence survivors has been highlighted by another study [43] as one of the most important barriers to access to health care for these vulnerable group. As reported by FGs/interviewees victims of violence may communicate a range of non-specific health problems in order to avoid revealing information about their traumatic experience. However, as emphasized by one study, clinical encounters are often complicated by inadequate communication between health professionals and patients [44]. Therefore, as suggested by another study [46], urgent training is required for practitioners and intercultural mediators to provide them with the skills needed for dealing with emotionally draining situations and to become familiar with international guidelines on providing care for victims of violence.

### Resource package development and dissemination

#### Content

FGs/interviewees indicated the core information and guidance that should be included in a RP. Primarily information was required regarding migrants’ entitlements to healthcare coverage including relevant legislative, financial and administrative issues impacting on healthcare accessibility. In particular the need to include guidelines to deal with particular vulnerable groups (e.g. unaccompanied minors, victims of violence) was emphasized. Since many health providers worked in very poorly organized settings and reception centres, they stressed the need to obtain information on the availability and distribution of specific health services, such as vaccinations, mental health, SRH, victims of violence care, and resources from other sectors (e.g.: housing, schooling, etc.). In order to help them overcome linguistic and cultural barriers, they underlined the importance of information on available interpreting services and specific tools to facilitate medical consultations. Guidance on intercultural competence training for health professionals, managers and administrative staff and specific issues concerning the health needs of migrants and the health care responses was highlighted as urgently needed in those countries most affected by massive arrivals (e.g.: Greece) and those countries that were relatively new to immigration influxes, (e.g.: Hungary, Slovenia). Finally, according to FGs/interviews respondents a RP should contain guidance for the design and implementation of a system to monitor migrants entering the health care system.

#### Format

Participants identified different methods of dissemination for the RP, but agreed that each country should choose the format best suited to existing strategies at national and local level. To this end, formats that facilitate interaction and engagement were preferred, such as training sessions, forums and workshops, rather than the use of written materials, web pages or brochures. The RP aimed at helping raise awareness and knowledge of health care providers about the barriers refugees and migrants encounter in seeking care and addressing these barriers with effective tools and measures. Therefore, it was reported, that a RP needed to favour the process of implementing solutions rather than simply passing on information about existing models.

#### Targeted users

Differing users of a RP were identified in order to maximize its impact. Most of the participants agreed that the RP should be addressed not only to front line professionals but also and above all to managers and those who are in a position to decide on the allocation of resources and on the possible implementation of the reported interventions. On the one hand, the various actors directly involved in the provision of health services, both governmental and non-governmental, as well as the operators of humanitarian organizations, would be able to benefit from the RP in their daily practice, on the other, the decision makers and service managers would be able to select sustainable measures and supervise their implementation.

#### Dissemination strategy

With regard to the most effective strategies for distributing the RP, participants argued that it would be useful to intervene at various levels: policies, organizations and communities. This systemic and integrated approach could, in their opinion, favour not only the dissemination of good practices, but also the creation of alliances, synergies, and the planning of shared interventions both at national and local level. For example, at policy level government agencies could include the RP between existing national training programs, migrant reception plans, and communication strategies. Similarly, health care organisations, and NGOs could disseminate the RP in reception centres, as well as in hospital and primary care settings. Finally, at community level, round tables for inter-sectoral committees and services could play a crucial role.

## Discussion

Our findings show that healthcare providers across Europe face different challenges in providing care for refugees and migrants at each stage of the migration trajectory: arrival, transit and destination. These challenges have an impact on healthcare coverage and accessibility for unauthorised entrants seeking asylum as well as for routine and irregular migrants. The legal status linked to the migration trajectory stage as well as the outcome of the asylum-seeking procedure play an important role in migrant’s access to healthcare. Lack of inclusive policies and effective administrative procedures to obtain entitlement appeared as the main barriers to accessing healthcare for refugees and migrants. Consequently, affordability is a second important barrier for those who are not in the position to enjoy full entitlement [7]. This is more problematic in insurance-based systems where the registration process may be particularly complicated, more so than in tax-funded systems. Although the most vulnerable groups of migrants (e.g.: children, pregnant women, victims of violence …) enjoy exemption from restrictions in many countries, our results show that barriers remain to accessing specific health services, such as specialist psychological and mental care; women care; child care and victim of violence care. Reasons for that vary across countries and are often determined by the discrepancies between what the law says and what is implemented in practice. One of the reasons is that unfamiliar procedures and the lengthy forms required to obtain exemption fees turn out to be barriers to health services even for these vulnerable groups [11]. Other reasons are, on the one hand, the lack of information on healthcare rights and service availability by migrants [47], and on the other hand, the lack of knowledge on legal issues by healthcare workers, who ignore the legal framework and the respective entitlements of these vulnerable groups.

Other barriers affecting refugees and migrants are a major responsibility of the healthcare organisations and systems that are still lacking responsive services and processes, such as interpretation and cultural mediation services; systems to collect information on the illness and health history of migrant patients; knowledge about entitlements and available services both on the part of migrants and healthcare providers; service organisation; and coordination between different providers. Despite considerable evidence that linguistic and cultural barriers are among the biggest obstacles in providing comprehensive and quality healthcare for migrants, in many countries the availability of professional interpreters or intercultural mediators is still limited because of lack of government policies and subsidies. Therefore, the introduction of a sufficient number of professional interpreters and intercultural mediators and their integration into existing organisational routines is envisaged. Our findings have identified different strategies concerning the implementation of intercultural mediation and interpreting services, that have also been summarized in recent literature [48]. Lack of reliable information on the illness and health history of patients for healthcare providers along the whole migration journey has been identified as another important challenge that needs urgent solutions. The pilot implementation of the electronic personal health record (ePHR) developed by IOM with the support of the European Commission has yielded fruitful results, however further research on its effectiveness and feasibility is needed.

How services are organised and delivered has been identified as an important barrier to accessing healthcare. Limited availability of services, unequal distribution of facilities, lack of transportation and complex referral systems have been described as important challenges during the refugee crisis. However, solutions that are only aimed at responding to emergencies have often led to fragmented and chaotic interventions, devolving attention from the need to develop structural changes in the EU health systems. To achieve this goal the strategies and tools developed by SH-CAPAC provide support for EU countries.

### Implications for health systems

This study aimed at collecting evidenced based information for the development of a RP containing a series of information, guidance and tools to support different actors providing care for refugees and migrants. However, since the context in which healthcare providers and decision makers operate is different from one country to another, information on proposed measures and resources to support access to healthcare should be adapted to local needs and integrated into the different means of communications. Thus, the solutions presented in this paper are to be seen as support measures for the development and dissemination of resource tools at country/regional/local level.

### Limitations

This study has certain limitations. Firstly, as it has been conducted within the time constraints of the SH-CAPAC project which was undertaken between January 1st and December 31st, 2016 under a European Commission emergency call for proposals in response to the refugee crisis in Europe. During the mere two months available to conduct interviews and FGs it was not possible to identify expert researchers in all EU countries, preventing us from collecting data from Germany, although they received the highest number of asylum applicants in the 2015 refugee crisis, and from including policy decision makers in the FGs/interviews. A further limitation arises from the fact that we used our own networks to approach interview/FG participants, and such purposeful sampling may have biased our findings. Nevertheless, without these networks we would never have been able to conduct this study in such a short period of time.

## Conclusion

Healthcare providers face important challenges in providing care for refugees and migrants and risk not being able to ensure equal access to quality care for these vulnerable groups. Access to healthcare is often hampered by the absence of inclusive legislation and policies and inadequate adjustment of health systems to the needs of these vulnerable populations. In particular, there is a need to improve the entitlement coverage of healthcare for the most vulnerable migrants and to carry out changes in administrative procedures, ensuring vital information for migrants and staff on rights to healthcare and promoting advocacy actions to drive national government policies, as well as guaranteeing coordination of the different partners involved in the provision of social and health services for migrants and refugees. To achieve this goal, it is crucial to frame migration as a permanent feature of the European social landscape rather than an emergency issue.

## Additional files

Additional file 1: Focus group/interview guide. (DOCX 33 kb)

Additional file 2: List of complete references included in the systematic review. (DOCX 113 kb)

Additional file 3: List of references included in the systematic review divided by barriers. (DOCX 58 kb)

## Data Availability

Other data and material are available upon request from the corresponding author.

## Abbreviations

A&E: Accident and emergency
EU: European Union
HEN: Health Evidence Network
IFRCRC: International Federation of Red Cross and Red Crescent Societies
IOM: International Organization for Migration
LHA: Local health authority
MdM: Médecins du Monde (Doctors of the World)
MI: Ministry of Interior
MOH: Ministry of Health
MS: Member State
MSF: Médecins sans Frontières (Doctors without Borders)
NGO: Non-Governmental Organization
OECD: Organisation for Economic Co-operation and Development
PRISMA: Preferred Reporting Items for Systematic Reviews and Meta-Analyses
SGBV: Sexual and gender-based violence
SRH: Sexual and reproductive health
UN: United Nations
UNHCR: United Nations High Commissioner for Refugees
WHO: World Health Organization

## Footnotes

1 The “resource package” for ensuring access to health care of refugees, asylum seekers and other migrants in the “European Union (EU) countries” was developed by the Azienda Unità Sanitaria Locale – IRCCS di Reggio Emilia, responsible for WP 4 of the EU project SH-CAPAC (http://www.sh-capac.org/) funded by CHAFEA (The Consumers, Health, Agriculture and Food Executive Agency) of the European Commission.

2 The COST Action ADAPT (Adapting European Health Systems to Diversity), an EU research network of experts from 30 countries; and the TF MED (Task Force on Migration, Equity and Diversity), a thematic group of the international network HPH (Health Promoting Hospitals and Health Services comprising healthcare providers from 16 countries.

3 http://www.observatoriodesigualdadandalucia.org/iniciativas/plataforma-somos-migrantes.
