# Supplementary material for: Supporting access to healthcare for refugees and migrants in European countries under particular migratory pressure
Source: BMC Health Serv Res. 2019 Jul 23;19:513. doi: 10.1186/s12913-019-4353-1 (PMC6651950; doi:10.1186/s12913-019-4353-1)
Supplement: Supplementary file 1 — Focus group/interview guide. (DOCX 33 kb) [file 12913_2019_4353_MOESM1_ESM.docx]

Supplementary file 1: FOCUS GROUP/INTERVIEW GUIDE

**Target groups**: professionals and managers involved in health care provision for refugees and asylum seekers – 8 to 12 participants for FG or face-to-face interview

**Inclusion criteria of the participants**

Health service providers and managers:

working at centers for refugees/AS, working in health services (primary care services, hospitals, health promotion/prevention) where AS go for health care, “hot spots” (persons intervening in “non-official” settlements), arrival camps (e.g. camps in border countries or landing countries), transit camps, destination centers, mental health services (general or specialized), physicians, nurses, psychologists, intercultural mediators/patient navigators/community health workers, services specialized in health care for victims of sexual violence, mother and child care, social workers (inpatient or outpatient services), volunteers for NGO, persons in charge of health services / head of health services / public officers in charge of health issues / refugees affairs at municipal/regional/national levels, civil servants working with ministries involved in health/justice/immigration

**Introduction**

Introduce yourself self and explain that you are conducting the FG/interview as part of a research at AUSL of Reggio Emilia, Italy within the EU funded project SH-CAPAC. Obtain written or verbal consent for audio-recording prior conducting the FG/interview (Use informed consent form).

**Presentation of the project to the participants**

This project aims at supporting member states to implement a coherent national and international response to the health needs of asylum seekers and refugees in Member States. In this work package, we aim at identifying available evidence on effective measures and tools in this context of the refugee crisis. To achieve this objective, we will develop a “resource package” to address barriers to health care for asylum seekers and refugees, disseminate it and support its implementation in member states.

**Round table of presentation**

Moderator asks each participant to briefly introduce herself/himself: profession, place of work, type of health service and experience with refugees or asylum seekers.

**First part: Challenges for health professionals and health care managers**

*Objective: to identify whether new barriers to health care services have emerged since the massive influx of asylum seekers in Europe.*

- What are the new challenges for your health service related to the current refugee crisis?
- How do these challenges impact your work as health professionals/health care managers?
- We now would like to discuss whether the situation of asylum seekers impact the challenges they face when accessing health services or when needing health care. Asylum seekers may be in their arrival phase, in a transition phase – waiting for a final destination or administrative procedures -, or in a permanent settlement phase, when they have finally reached their final destination. Do you perceive different challenges according to the situation of the asylum seekers?
- Are there any specific situations in which specific challenges arise? (e.g. mental health needs, mother & child care, chronic diseases, sexual health/violence issues, transnational care…)

**Part 2: Solutions and best practices**

*Objective: to identify best practices and the conditions that support their development*

- What are the new effective strategies that have been developed to face these new challenges?

*Exploration questions if necessary:*

*Could you detail the content and process behind the strategy?*

*Who was in charge of the strategy?*

*What were the expected outcomes of the strategy?*

*Did you reach these outcomes? If not, what hampered the strategy? If yes, what supported the strategy at organizational level?*

*Would you recommend this strategy to other organizations?*

*What would they need to implement it successfully?*

**Part 3: Development and dissemination of the resource package**

*Objective: to identify the required content, the adequate format and dissemination strategies for the resource package in participating countries*

This project aims at developing resource packages for health professionals, health care managers, health services and any actors involved in health care for asylum seekers and refugees across Europe.

- What should be the content of this resource package in order to support your practice as a health care professional / as a health care manager?
- What are the ideal formats for a resource package *(e.g. a website, an app’, a flyer, a brochure, e-learning course,…)* ? *Probe: What will be the most useful format for you? Note to the moderator: please ask each participant individually / round table*
- Which groups of health care professionals or health care managers are the most likely to maximize the impact of this resource package?
- What would be the most effective strategies to disseminate the resource package to all actors involved in this topic in your country?

**Part 4: Closing of the focus group or interview**

Thanks to the participants. Invite them for a last comment / remark on the project/ the discussion. Invite the participant to share any relevant material related to best practices or barriers with the research team.

PLEASE DO NOT FORGET to ask participants to leave their email addresses / phone numbers for further contacts and to receive the resource package (if they accept).

**Contact data of the local research expert**

[Name of the contact person from the research team]

Email address

**Contact data of the main investigator**

Antonio Chiarenza, AUSL of Reggio Emilia

Email address : antonio.chiarenza@ausl.re.it
