# Supplementary material for: Supporting access to healthcare for refugees and migrants in European countries under particular migratory pressure
Source: BMC Health Serv Res. 2019 Jul 23;19:513. doi: 10.1186/s12913-019-4353-1 (PMC6651950; doi:10.1186/s12913-019-4353-1)
Supplement: Supplementary file 2 — List of complete references included in the systematic review. (DOCX 113 kb) [file 12913_2019_4353_MOESM2_ESM.docx]

Supplementary file 2: list of complete references included in the systematic review

| **First author and title of the article** | **Year** | **Country** | **Objective of the study** | **Study population** | **methodology** |
| --- | --- | --- | --- | --- | --- |
| 1. Abbing, H.D., ***Age determination of unaccompanied asylum seeking minors in the European Union: a health law perspective.*** | 2011 | Europe | to create of EU best practice guidelines for age determination amongst unaccompanied asylum seeking minors. | Unaccompanied minors | qualitative |
| 1. Ahmad, F., et al., ***A pilot with computer-assisted psychosocial risk-assessment for refugees.*** | 2012 | Canada | to create interactive eHealth tools (multi-risk Computer-assisted Psychosocial Risk Assessment CaPRA) that could build bridges between medical and social care in a timely manner. | Afghan refugees | quantitative |
| 1. Ahmed, A., et al., ***Experiences of immigrant new mothers with symptoms of depression.*** | 2008 | Canada | to better understand immigrant new mothers with depressive symptoms (a) experiences and attributions of depressive symptoms, (b) their experiences with health care providers and support services, (c) factors that facilitated or hindered help seeking, (d) factors that aided recovery or (e) were associated with women continuing to experience symptoms of depression. to identify barriers and to offer suggestion for improvement. | refugees, asylum seeking, non-refugee, and immigrant women | qualitative |
| 1. Akar, F.A., et al., ***The Istanbul protocol (manual on the effective investigation and documentation of torture and other cruel, inhuman or degrading treatment or punishment): implementation and education in Israel.*** | 2014 | Israel | to standardize the implementation of rules concerning (domestic) violence against women, children and the elderly, the management of cases where patients have been subjected to violence while under the custody of legal enforcement agencies, or patients who have been victims of torture. To implement a manual on the effective investigation and documentation of torture and other cruel, inhuman or degrading treatment or punishment. | refugees and asylum seekers women, children and elders | qualitative |
| 1. Al-Obaidi, A., et al., ***Incorporating Preliminary Mental Health Assessment in the Initial Healthcare for Refugees in New Jersey.*** | 2015 | United States | to assess the feasibility of introducing a mental health screening tool into the initial health care assessment for refugees in New Jersey, US. | refugees | qualitative |
| 1. Alayarian, A., ***Children, torture and psychological consequences.*** | 2009 | United Kingdom | to provide clinical services, to influence policy and practice by searching for evidence and demonstrating solutions to improve the lives, homes and communities of children disadvantaged by torture and the services that support them and to provide some remedies to children of refugees who are suffering the consequence of trauma that they experienced and demonstrate good practice. | refugee children | qualitative |
| 1. Anders, A.D.P. and J.N. Lester, ***Navigating authoritarian power in the United States: Families with refugee status and allegorical representation.*** | 2015 | United States | to examine the cultivation of modern convictions in the elementary school and health care system, and the influence of such convictions at the intersection of authoritarian power. | Burundian refugees | qualitative |
| 1. Asgary, R. and C.L. Smith, **Ethical and professional considerations providing medical evaluation and care to refugee asylum seekers.** | 2013 | United States | to review ethical concerns in regard to accountability, the patient-physician relationship, and moral responsibilities to offer health care irrespective of patient legal status; competing professional responsibility toward society and the judiciary system; concerns about the consistency of asylum seekers' claims; ethical concerns surrounding involving trainees and researching within the evaluation setting; and the implication of broader societal views towards rights and social justice. | asylum seekers | literature review |
| 1. Asgary, R. and N. Segar, ***Barriers to health care access among refugee asylum seekers.*** | 2011 | United States | to portray the access to health care of asylum seekers. | asylum seekers | qualitative |
| 1. Aspinall, P., ***Vulnerable Migrants, Gypsies and Travellers, People Who Are Homeless, and Sex Workers: A Review and Synthesis of Interventions/Service Models that Improve Access to Primary Care & Reduce Risk of Avoidable Admission to Hospitals*** | 2014 | United Kingdom | to provide a foundation for understanding the changes that might need to be brought about in the health and related systems to meet the needs of individuals living in an unequal society. | asylum seekers, undocumented migrants, refugees and migrants | literature review |
| 1. Baarnhielm, S., C. Javo, and M.O. Mosko, ***Opening up mental health service delivery to cultural diversity: current situation, development and examples from three northern European countries.*** | 2013 | Germany, Norway, Sweden | to analyse the barriers to mental health care access for refugees, migrants and minorities, and problems with quality of culturally sensitive care in the three countries. | refugees, migrants and minorities | literature review |
| 1. Baarnhielm, S., et al., **Approaching the vulnerability of refugees: evaluation of cross-cultural psychiatric training of staff in mental health care and refugee reception in Sweden.** | 2014 | Sweden | to evaluate the outcomes of cross-cultural mental health training given to professionals in health care and refugee reception in Stockholm, Sweden. | health care professionals, refugee reception professionals | mixed approach |
| 1. Baird, M.B., ***Well-being in refugee women experiencing cultural transition.*** | 2012 | United States | to present a situation-specific theory of well-being in refugee women experiencing cultural transition. | South-Sudanese refugee women | qualitative |
| 1. Balaam, M.C., et al., ***A qualitative review of migrant women's perceptions of their needs and experiences related to pregnancy and childbirth.*** | 2013 | INT | to synthesis the evidence related to migrant women's perceptions of their needs and experiences in relation to pregnancy and childbirth. | refugee and immigrant women | literature review |
| 1. Balaam, M.C., et al., ***'We make them feel special': The experiences of voluntary sector workers supporting asylum seeking and refugee women during pregnancy and early motherhood.*** | 2016 | United Kingdom | to explore the experiences of voluntary sector workers supporting asylum seeking and refugee women during pregnancy and early motherhood. | asylum seeking and refugee pregnant women | qualitative |
| 1. Balachandra, S.K., et al., ***Family-centered maternity care for deaf refugees: the patient-centered medical home in action.*** | 2009 | United States | to apply principles of the patient-centered medical home - PCMH to address an extremely challenging clinical situation: providing high-quality maternity care to a recently immigrated Vietnamese refugee couple lacking formal language skills. | Vietnamese refugee couples, deaf Vietnamese refugees | qualitative |
| 1. Bell, P. and E. Zech, ***Access to mental health for asylum seekers in the European union: An analysis of disparities between legal rights and reality.*** | 2009 | Belgium | to explore some of the issues surrounding access to mental health care for asylum seekers, using Belgium as a case in point and to address the discrepancies that continue to exist between member states, notably policies on health care for refugees, and in particular mental healthcare. | asylum seekers | qualitative |
| 1. Bellamy, K., et al., ***Access to medication and pharmacy services for resettled refugees: a systematic review.*** | 2015 | NR | to review systematically the literature and synthesise findings of research that explored barriers and/or facilitators of access to medication and pharmacy services for resettled refugees. | refugees | literature review |
| 1. Beltran-Avery PP. ***'The role of the health care interpreter’***, National Council on Intepreting in Health Care. 2011. | 2011 | United States | to explore the evolution of the role of the health care interpreter. | stakeholders | qualitative |
| 1. Bennett, S. and J. Scammell, ***Midwives caring for asylum-seeking women: research findings.*** | 2014 | United Kingdom | to explore the experiences of midwives caring for asylum seeking women. | asylum seeking women | qualitative |
| 1. Berthold, S.M., et al., ***Comorbid mental and physical health and health access in Cambodian refugees in the US.*** | 2014 | United States | to identify the relationship between mental and physical health problems and barriers to healthcare access in Cambodian refugee adults. | Cambodian refugees | quantitative |
| 1. Bischoff , A., et al. ***Doctor – Patient Gender Concordance and Patient Satisfaction in Interpreter-Mediated Consultations: An Exploratory Study.*** | 2008 | Switzerland | to explore the effect of doctor – patient gender concordance on satisfaction of foreign language – speaking patients in consultations with and without a professional interpreter. | refugees and immigrants | quantitative |
| 1. Bischoff, A. and K. Denhaerynck, ***What do language barriers cost? An exploratory study among asylum seekers in Switzerland.*** | 2010 | Switzerland | to investigate the association between language barriers and the costs of health care. | asylum seekers | quantitative |
| 1. Bodenmann, P. and A.R. Green, ***Health disparities: Local realities and future challenges.*** | 2012 | Switzerland | to describe the potential disparities taht vulnerable population face in order to explain their cause, and propose possible solutions. | asylum seekers, undocumented immigrants, marginalised Swiss natives and immigrant communities | qualitative |
| 1. Bogenschutz, M., ***"We find a way": challenges and facilitators for health care access among immigrants and refugees with intellectual and developmental disabilities.*** | 2014 | United States | to discover the particular challenges that immigrants with disabilities face when accessing health care, and the facilitating factors that assist them in this process. | disabled refugees and immigrants | qualitative |
| 1. Boise, L., et al., ***African refugee and immigrant health needs: report from a community-based house meeting project.*** | 2013 | United States | to gather data about the perceived health needs and barriers to health care Africans encounter, and lay the foundation for a program of action to guide APH's future work. | African refugees & immigrants | qualitative |
| 1. Borland, R. and C. Zimmerman, ***Caring for trafficked persons. Guidance for health professionals.*** | 2012 | INT | to provide practical, non-clinical guidance to help concerned health providers understand the phenomenon of human trafficking, recognize some of the health problems associated with trafficking and consider safe and appropriate approaches to providing health care for trafficked persons. It outlines the health provider’s role in providing care and describes some of the limitations of his or her responsibility to assist. | refugees and asylum seekers | mixed approach |
| 1. Borland, R. and C. Zimmerman, ***Caring for trafficked persons. Training facilitator's guide.*** | 2012 | INT | to present a facilitator’s guide and accompanying materials for individuals who wish to carry out training for health providers. | refugees and asylum seekers | qualitative |
| 1. Boynton, L., et al., ***The role of stigma and state in the mental health of Somalis.*** | 2010 | United States | to present a case report of a 55-year-old Somali refugee suffering from depression and posttraumatic stress disorder. | Somali refugees | qualitative |
| 1. Bradby H, Humphris R, Newman P, Phillimore J. ***Public health aspects of migrant health: a review of the evidence on health status for refugees and asylum seekers in the European Region***. Health Evidence Network synthesis report. 2015 | 2015 | INT | To review available evidence and examining which policies and interventions would work to improve accessibility and quality of health care delivery for asylum seekers and refugees. | health status for refugees and asylum seekers in the European Region | mixed approach |
| 1. Bradby, H., et al.**Refugees and asylum seekers in the European Region - reviewing the research evidence.** | 2016 | Europe | to identify which policies and interventions work to improve health care access and delivery for asylum seekers and refugees in the European Region. | refugees and asylum seekers | literature review |
| 1. Bradby, H., et al., ***Public health aspects of migrant health: a review of the evidence on health status for refugees and asylum seekers in the European Region*** | 2015 | INT | to synthesize research findings from a systematic review of available academic evidence and grey literature to address the following question: what policies and interventions work to improve health care access and delivery for asylum seekers and refugees in the European Region? | refugees and asylum seekers | literature review |
| 1. Brandon Chen, Y.Y., et al., ***Improving access to mental health services for racialized immigrants, refugees, and non- status people living with HIV/AIDS.*** | 2015 | Canada | to explore IRN- PHAs’ (people living with HIV AIDS) mental health service- seeking behaviours, service utilization experiences, and give suggestions for service improvements. | refugees, immigrant, and non- status migrants | qualitative |
| 1. Briones-Vozmediano, E., et al. ***Barriers and facilitators to effective coverage of Intimate Partner Violence services for immigrant women in Spain.*** | 2015 | Spain | to explore service providers' perceptions in order to identify barriers and facilitators to effective coverage of Intimate Partner Violence (IPV) services for immigrant women in Spain, according to the different categories proposed in Tanahashi's model of effective coverage. | refugee and immigrant women | qualitative |
| 1. Briscoe, L,. & Lavender, T. **Exploring maternity care for asylum seekers and refugees. .** | 2009 | United Kingdom | to explore and synthesize the experience of maternity care by female asylum seekers and refugees. | refugees and asylum seekers | qualitative |
| 1. Brolan, C.E., et al., ***Invisible populations: parallels between the health of people with intellectual disability and people of a refugee background.*** | 2011 | Australia | to recognise the importance of health policy in leading affirmative action to ensure these populations become visible in the implementation of the National Primary Health Care Strategy. | refugees, humanitarian entrants and people with intellectual disability | qualitative |
| 1. Brown, E., et al., ***"They get a C-section...they gonna die": Somali women's fears of obstetrical interventions in the United States.*** | 2010 | United States | to explore sources of resistance to common prenatal and obstetrical interventions among 34 Somali resettled adult women in Rochester, New York. | Somali refugee women | qualitative |
| 1. Burchill J. ***Safeguarding vulnerable families: work with refugees and asylum seekers.*** | 2011 | United Kingdom | to explore the experiences of health visitors working with refugees and asylum seekers. | health visitors, refugee families | qualitative |
| 1. Campbell, R., et al., ***A Comparison of Health Access Between Permanent Residents, Undocumented Immigrants and Refugee Claimants in Toronto, Canada.*** | 2014 | Canada | to examine the vulnerabilities of undocumented immigrant and contrast their experiences seeking healthcare with refugee claimants and permanent residents. | asylum seekers, undocumented migrants and migrants | qualitative |
| 1. Charbonneau, C.J., et al. ***Exploring the views of and challenges experienced by dental hygienists practising in a multicultural society: A pilot study.*** | 2014 | Canada | to explore the views of and challenges experienced by dental hygienists practising in a multicultural society. | refugees, new immigrants, Aboriginal people, and people of low economic status | qualitative |
| 1. Chauvin, P., et al. ***Non access to vaccinations among migrant and ethnic minorities’ children***: analysis from Doctors of the World International Network Observatory. | 2016 | Europe | to collect data on immunization among children and to identify barriers to immunization. | refugee children | qualitative |
| 1. Chauvin, P., et al., ***Access to healthcare for people facing multiple vulnerabilities in health.*** | 2015 | Europe | to describe the epidemiological situations of vulnerable migrant groups and their barriers when accessing health care services. | refugees and asylum seekers | qualitative |
| 1. Cheng, I.H., A. et al. ***Refugee experiences of general practice in countries of resettlement: a literature review.*** | 2015 | United Kingdom | to describe and analyse the literature on the experiences of refugees and asylum seekers using general practice services in countries of resettlement. | refugees and asylum seekers | literature review |
| 1. Cheng, I.H., et al., ***Importance of community engagement in primary health care: the case of Afghan refugees.*** | 2015 | Australia | to describe how the Afghan pre-migration experiences of primary health care can affect engagement with Australian primary care services, including the implications for Australian primary health care policy, planning and delivery. | Afghan refugees | qualitative |
| 1. Cheng, I.H., et al., ***Rites of passage: improving refugee access to general practice services.*** | 2015 | Australia | to analyse the factors influencing Afghan refugees' access to general practice. | Afghan refugees | qualitative |
| 1. Chiumento, A., et al., ***School-based mental health service for refugee and asylum seeking children: multi-agency working, lessons for good practice.*** | 2011 | United Kingdom | to describe the Haven Project: a school based Child and Adolescent Mental Health Service (CAMHS) for refugee children in Liverpool and to present a multiagency model for replication across community mental health services. | refugee children and adolescents | qualitative |
| 1. Clark, A., et al., ***'Excuse me, do any of you ladies speak English?' Perspectives of refugee women living in South Australia: barriers to accessing primary health care and achieving the Quality Use of Medicines.*** | 2014 | Australia | to identify the barriers to accessing primary health care services and explore medicine-related issues as experienced by refugee women in South Australia. | Sudanese, Burundese, Congolese, Burma, Afghan and Bhutanese refugee women | qualitative |
| 1. Cobb, T.G., ***Strategies for providing cultural competent health care for Hmong Americans.*** | 2010 | United States | to enumerate the barriers to providing health care to Hmong Americans and share strategies to respect Hmong culture when providing quality health care. | Hmong refugees | qualitative |
| 1. Colucci, E., et al., ***In or out? Barriers and facilitators to refugee-background young people accessing mental health services.*** | 2015 | Australia | to explores barriers and facilitators to engaging young people from refugee backgrounds with mental health services | youth refugees | qualitative |
| 1. Colucci, E., et al., ***The utilisation of mental health services by children and young people from a refugee background: a systematic literature review.*** | 2014 | NR | to summarize what is known about the use of mental-health services by children and young people of refugee background and to identify factors that may constitute impediments to service use as well as factors that may facilitate access to and engagement with services. | children and young refugees | literature review |
| 1. Correa-Velez, I. and J. Ryan, ***Developing a best practice model of refugee maternity care.*** | 2012 | Australia | to develop a best practice model of maternity care for women from refugee backgrounds at a major maternity hospital in Brisbane, Australia. | pregnant refugee women | mixed approach |
| 1. Crosby, S.S., ***Primary care management of non-English-speaking refugees who have experienced trauma: a clinical review.*** | 2013 | INT | to discuss the importance of and methods for obtaining refugee trauma histories, to recognize the psychological and physical manifestations of trauma characteristic of refugees, and to explore how cultural differences and limited English proficiency affect the refugee patient-clinician relationship and how to best use interpreters. | refugees | literature review |
| 1. Degni, F.,et al. **Communication and Cultural Issues in Providing Reproductive Health Care to Immigrant Women: Health Care Providers' Experiences in Meeting Somali Women** Living in Finland. | 2011 | Finland | to explore physicians-nurses/midwives' communication when providing reproductive and maternity health care to Somali women in Finland. | Somali refugee women | qualitative |
| 1. Derluyn, I. and E. Broekaert, ***Unaccompanied refugee children and adolescents: the glaring contrast between a legal and a psychological perspective.*** | 2008 | Belgium | to show the 'psychological' perspective and the necessity of a strongly elaborated reception and care system for these children and adolescents in order to meet their specific situation and needs. | unaccompanied minors | qualitative |
| 1. DeStephano, C.C., P.M. Flynn, and B.C. Brost, ***Somali prenatal education video use in a United States obstetric clinic: a formative evaluation of acceptability.*** | 2010 | United States | to explore the acceptability of health education videos by Somali refugee women in a clinical setting. | Somali refugee women | qualitative |
| 1. Drummond, P.D., et al., ***Barriers to accessing health care services for West African refugee women living in Western Australia.*** | 2011 | Australia | to survey help-seeking pathways and barriers to accessing health care services in 51 West African refugee women who had settled recently in Perth, and in 100 Australian women. | West African refugee women | quantitative |
| 1. Duguet, A.M. and B. Bévière, ***Access to health care for illegal immigrants: A specific organisation in France.*** | 2011 | France | to present the French system of social protection, the "Couverture médicale universelle" or CMU, which provides the same protection to asylum seekers and documented immigrants as to nationals, and the "Aide médicale d'état" or AME, that is open to every person who does not fulfil the legal conditions to obtain the CMU, such as illegal immigrants. | asylum seekers and documented immigrants | qualitative |
| 1. Duke, P. and F. Brunger, ***The MUN Med Gateway Project: marrying medical education and social accountability.*** | 2015 | Canada | to provide access to family physicians and continuity of care for newly arrived refugees; to provide opportunities for medical students to practise cross-cultural health care; and to mentor medical students in advocacy for underserved populations. | refugees | qualitative |
| 1. Dutcher, G.A., et al. ***The Refugee Health Information Network: a source of multilingual and multicultural health information.*** | 2008 | United States | to improve health services for refugees and asylums seekers. This is also a network designed to facilitate collaboration and sharing among state refugee health coordinators and clinics providing services to refugee and immigrant communities. | refugees and asylum seekers | qualitative |
| 1. Ellis, B.H., et al., ***New directions in refugee youth mental health services: Overcoming barriers to engagement.*** | 2011 | United States | to describe how Barriers like (a) distrust of authority and/or systems, (b) stigma of mental health services, (c) linguistic and cultural barriers, and (d) primacy and prioritization of resettlement stressors, may prevent refugee youth from receiving mental health services; To describe approaches to addressing them ; to describe of Supporting the Health of Immigrant Families and Adolescents (Project SHIFA), a program developed in collaboration with the Somali community in Boston, Massachusetts. | youth refugees | qualitative |
| 1. Elwell, D., et al., ***Refugees in Denver and their perceptions of their health and health care.*** | 2014 | United States | to assess the self-perceived health of and barriers to care for refugees in the Denver metro area in order to understand better the needs of this population | refugees | quantitative |
| 1. Fang, DM & Baker, DL. **Barriers and Facilitators of Cervical Cancer Screening among Women of Hmong Origin.** | 2013 | United States | to explore the barriers and facilitators of cancer screening among women of Hmong origin. | Hmong refugee women | qualitative |
| 1. Farokhi, M.R. et al. ***A student operated, faculty mentored dental clinic service experience at the University of Texas Health Science Center at San Antonio for the underserved refugee community: an interprofessional approach.*** | 2014 | United States | to create the student-run San Antonio Refugee Health Clinic (SARHC), that serves the refugees by providing free health care/education while connecting them to San Antonio's primary health care system. | refugees | qualitative |
| 1. Fatahi, N., et al., ***Experiences of Kurdish war-wounded refugees in communication with Swedish authorities through interpreter.*** | 2010 | Sweden | to study experiences of war-wounded Kurdish refugees with respect to cross-cultural communication through interpreters | war-wounded Kurdish refugees | qualitative |
| 1. Feldman, R., ***When maternity doesn’t matter Dispersing pregnant women seeking asylum*** | 2013 | United Kingdom | to investigate the health impact of dispersal and relocation on pregnant women and new mothers seeking asylum. | asylum seeking pregnant women | literature review |
| 1. Flynn, A. and D. Flynn, ***'Give us the weapon to argue': eHealth and the Somali community of Manchester.*** | 2008 | United Kingdom | to investigate the perceptions of a marginalised community, the Somali community in Manchester, UK, with regard to the possible benefits and disadvantages of eHealth as a means of providing patient healthcare information. | Somali refugees | qualitative |
| 1. Furber, S., et al., ***A qualitative study on tobacco smoking and betel quid use among Burmese refugees in Australia.*** J Immigr Minor Health, 2013. **15**(6): p. 1133-6. | 2013 | Australia | to explore the beliefs and experiences of Burmese refugees in Wollongong on smoking to guide the development of smoking cessation interventions. | Burmese men refugees | qualitative |
| 1. Furler, J., et al., ***Managing depression among ethnic communities: a qualitative study.*** Ann Fam Med, 2010. **8**(3): p. 231-6. | 2010 | Australia | to explores the complexities of this work through a study of how family physicians experience working with different ethnic minority communities in recognizing, understanding, and caring for patients with depression. | refugees and immigrant women | qualitative |
| 1. Gagnon, A.J., et al., ***Do referrals work? Responses of childbearing newcomers to referrals for care.*** J Immigr Minor Health, 2010. **12**(4): p. 559-68 | 2010 | Montreal | to explore the inhibitors and facilitators of migrant women for following through with referrals for care. | Refugee, asylum-seeker, and immigrant) women | qualitative |
| 1. Gele AA, Torheim LE, Pettersen KS, Kumar B. ***Beyond Culture and Language: Access to Diabetes Preventive Health Services among Somali Women in Norw****ay.* | 2015 | Norway | to analyse the Access to Diabetes Preventive Health Services among Somali Women in Norway. | refugees and immigrants | qualitative |
| 1. Geltman, P.L., et al., ***Health literacy, acculturation, and the use of preventive oral health care by Somali refugees living in massachusetts.*** J Immigr Minor Health, 2014. **16**(4): p. 622-30. | 2014 | USA | to investigate the impact of English health literacy and spoken proficiency and acculturation on preventive dental care use among Somali refugees in Massachusetts. | Somali refugees | quantitative |
| 1. Geltman, P.L., et al., ***The impact of functional health literacy and acculturation on the oral health status of somali refugees living in Massachusetts.*** American Journal of Public Health, 2013. **103**(8): p. 1516-1523. | 2013 | USA | to assess the impact of health literacy and acculturation on oral health status of Somali refugees in Massachusetts. | Somali refugees | quantitative |
| 1. Gibbs, L., et al., ***An exploratory trial implementing a community-based child oral health promotion intervention for Australian families from refugee and migrant backgrounds: a protocol paper for Teeth Tales.*** BMJ Open, 2014. **4**(3): p. e004260 | 2014 | Australia | to establish a model for child oral health promotion for culturally diverse communities in Australia. | Iraqi, Lebanese or Pakistani refugee families | quantitative |
| 1. Ginieniewicz, J. and K. McKenzie, ***Mental health of Latin Americans in Canada: a literature review.*** Int J Soc Psychiatry, 2014. **60**(3): p. 263-73. | 2014 | Canada | to review the literature on the mental health of Latin American immigrants to Canada and identify possible barriers. | Central American refugees | literature review |
| 1. Goosen, S., I.E. van Oostrum, and M.L. Essink-Bot, ***[Obstetric outcomes and expressed health needs of pregnant asylum seekers: a literature survey]***. | 2010 | The Netherlands | to analyse whether specific attention is needed for the improvement of health for pregnant asylum seekers by producing an overview of obstetric outcomes, risk factors and expressed health needs of asylum seekers. | asylum seekers | literature review |
| 1. Graham, E.A., et al., ***Health services utilization by low-income limited English proficient adults.*** | 2008 | United States | to evaluate the health care utilization of limited English proficiency (LEP) compared to English proficient (EP) adults with the same health insurance (Medicaid managed care) and full access to professional medical interpreters. | refugees and immigrants | quantitative |
| 1. Grant, K.J., et al., ***The refugee experience of acquiring a family doctor.*** | 2015 | Canada | to explore refugees' experiences of the barriers and facilitators involved in finding a regular family doctor. | Iranian, Afghan, Myanmar, Vietnamese, and Latino-american refugees | qualitative |
| 1. Grazier, K.L., ***Integrating behavioral health care and primary care: Application of a clinical and economic model in culturally diverse communities.*** | 2008 | United States | to integrate behavioural health care and primary care. | refugees, immigrants, and other groups vulnerable | qualitative |
| 1. Grigg-Saito, D., et al., ***Building on the strengths of a Cambodian refugee community through community-based outreach.*** | 2008 | United States | to eliminate health disparities in cardiovascular disease and diabetes. | Cambodian refugees | qualitative |
| 1. Grigg-Saito, D., et al., ***Long-term development of a "whole community" best practice model to address health disparities in the Cambodian refugee and immigrant community of Lowell, Massachusetts.*** | 2010 | United States | to overcame health disparities. | Cambodian refugees | quantitative |
| 1. Gudeva Nikovska, D., et al. **Health services for migrants on the Balkan route - is Macedonia up to the challenge?** | 2016 | Macedonia | to assess current health situation in the 2 transit centers, identify health related activities in the project area, availability of health care services for the target populations and map actors involved in humanitarian and health assistance. | refugees | qualitative |
| 1. Gurnah, K., et al., ***Lost in Translation: Reproductive Health Care Experiences of Somali Bantu Women in Hartford, Connecticut.*** | 2011 | United States | to explore the reproductive health experiences of 1 such population-Somali Bantu women in Connecticut-to identify potential barriers to care experienced by marginalized populations | Somali Bantu refugee women | qualitative |
| 1. Hackett, J., et al., ***Evaluation of three population health capacity building projects delivered by videoconferencing in NSW.*** | 2009 | United Kingdom | to evaluate three population health capacity building projects. | refugees | qualitative |
| 1. Hadgkiss, E.J. and A.M.N. Renzaho, ***The physical health status, service utilisation and barriers to accessing care for asylum seekers residing in the community: a systematic review of the literature.*** | 2014 | Australia | to document physical health problems that asylum seekers experience on settlement in the community and to assess their utilisation of healthcare services and barriers to care, in an international context. | asylum seekers | literature review |
| 1. Haith-Cooper, M. and G. Bradshaw, **Meeting the health and social care needs of pregnant asylum seekers;** midwifery students' perspectives: part 3; "the pregnant woman within the global context"; an inclusive model for midwifery education to address the needs of asylum seeking women in the UK. | 2013 | United Kingdom | to describe the conceptualisation and development of an inclusive educational model. | pregnant asylum-seeking women | literature review |
| 1. Haley, H.L., et al., ***Primary prevention for resettled refugees from Burma: where to begin?*** | 2014 | United States | to develop effective primary prevention initiatives to help recently arrived refugees retain some of their own healthy cultural habits and reduce the tendency to adopt detrimental ones | Burma refugees | qualitative |
| 1. Harstad, I., et al., ***Screening and treatment of latent tuberculosis in a cohort of asylum seekers in Norway.*** | 2010 | Norway | to assess follow-up of screening results at different healthcare levels in relation to demographics, screening results and organizational factors, and how this influenced treatment of latent tuberculosis. | asylum seekers | quantitative |
| 1. Hassan, G., et al., ***Mental health and psychosocial wellbeing of Syrians affected by armed conflict.*** | 2016 | NR | to provide information on cultural aspects of mental health and psychosocial wellbeing relevant to care and support for Syrians affected by the crisis. | Syrians refugees | literature review |
| 1. Hauck, F.R., et al., ***Factors Influencing the Acculturation of Burmese, Bhutanese, and Iraqi Refugees Into American Society: Cross-Cultural Comparisons.*** | 2014 | United States | to examine the factors influencing the acculturation of Burmese, Bhutanese, and Iraqi Refugees in the United States. | Burmese, Bhutanese, and Iraqi refugees | qualitative |
| 1. Haworth, R.J., et al., ***Knowledge, attitudes, and practices for cervical cancer screening among the Bhutanese refugee community in Omaha, Nebraska.*** | 2014 | United States | to investigate cervical cancer and screening knowledge and perceptions about the susceptibility and severity of cervical cancer and perceived benefits and barriers to screening. | Bhutanese refugee women | mixed approach |
| 1. Helweg-Larsen, M. and L.M. Stancioff, ***Acculturation matters: risk perceptions of smoking among Bosnian refugees living in the United States.*** | 2008 | United States | to investigate acculturation and risk perceptions of heart attack and lung cancer among a group of refugees. | Bosnian refugees | quantitative |
| 1. Henley, J. and J. Robinson, ***Mental health issues among refugee children and adolescents.*** | 2011 | Australia | to raise awareness of mental health issues for refugee children, empowering clinicians to engage effectively with this client group. | children and adolescents refugees | literature review |
| 1. Higginbottom, G.M., et al., ***"I have to do what I believe": Sudanese women's beliefs and resistance to hegemonic practices at home and during experiences of maternity care in Canada.*** | 2013 | Canada | to analyse difficulties in difficulty in access to maternity care services. | Sudanese pregnant refugee women | qualitative |
| 1. Hill, L., et al., ***Inter-professional learning to prepare medical and social work students for practice with refugees and asylum seekers.*** | 2009 | United Kingdom | to describe the genesis and implementation of a series of innovative inter-professional workshops for medical and social work students, focussing specifically on marginalised groups. | refugees and asylum seekers | qualitative |
| 1. Hjern, A., et al. ***Age assessment of young asylum seekers.*** | 2012 | Europe | to describe the difficulties for age assessment of young asylum seekers. | young asylum seekers | qualitative |
| 1. Hudelson, P.et al. **Quality in practice: integrating routine collection of patient language data into hospital practice**. | 2013 | Switzerland | to explore the feasibility and acceptability of a procedure for collecting patient language data at the first point of contact, prior to its hospital-wide implementation. | refugees | quantitative |
| 1. Hughes, G., ***Finding a voice through 'The Tree of Life': a strength-based approach to mental health for refugee children and families in schools.*** | 2014 | United Kingdom | to overcame the difficulties of access to traditional mental health services. | refugees families | qualitative |
| 1. Iliadi, P., ***Refugee women in Greece: - a qualitative study of their attitudes and experience in antenatal care.*** | 2008 | Greece | to examine whether refugee women, resettled in Greece, receive antenatal care and to explore possible factors that may influence their attitude towards maternal care. | refugee women | qualitative |
| 1. Im, H. and R. Rosenberg, ***Building Social Capital Through a Peer-Led Community Health Workshop: A Pilot with the Bhutanese Refugee Community.*** | 2016 | United States | to assess the impact of a pilot peer-led community health workshop (CHW) in the Bhutanese refugee community. | Bhutanese refugees | qualitative |
| 1. Ingram, J., ***The health needs of the Somali community in Bristol.*** | 2009 | United Kingdom | to identify the health needs of the Somali community in Bristol. | Somali refugees | qualitative |
| 1. International Organization for Migration, ***International Migration, Health and Human Rights*** | 2013 | INT | to devote particular attention to the most vulnerable categories of migrants and conceptualizes vulnerability as directly resulting from an inherent characteristic of the individual migrant or group (e.g. gender, age, disability, HIV status, lack of safety net and poor education) and as related to its fundamental structural causes (e.g. working and living conditions; lack of legal protection, including that in relation to the migrant’s legal status in the host country; crime and conflict; language and cultural barriers; lack of formal and informal social protections offered during and after the migration process; and immigration detention). | asylum seekers, undocumented migrants, refugees and migrants | literature review |
| 1. Ioannidi, E. **First reception of refugees entering through the Aegean. The current situation in Greek islands.** | 2015 | Greece | to describe challenges in providing care and access to health care in Greek islands. | asylum seekers | qualitative |
| 1. IOM, **Bulgaria** | 2015 | Bulgaria | to present the results of the assessment of migrant, occupational, and public health which took place in Bulgaria within the framework of the IOM Equi Health project. | refugees and asylum seekers | mixed approach |
| 1. IOM, **Croatia** | 2015 | Croatia | to present the results of the assessment of migrant, occupational, and public health which took place in Croatia within the framework of the IOM Equi Health project. | refugees and asylum seekers | mixed approach |
| 1. IOM, **Spain** | 2015 | Spain | to present the results of the assessment of migrant, occupational, and public health which took place in Spain within the framework of the IOM Equi Health project . | refugees and asylum seekers | mixed approach |
| 1. IOM,**Greece** | 2015 | Greece | to present the results of the assessment of migrant, occupational, and public health which took place in Greece within the framework of the IOM Equi Health project. | refugees and asylum seekers | mixed approach |
| 1. IOM,**Italy** | 2015 | Italy | to present the results of the assessment of migrant, occupational, and public health which took place in Italy within the framework of the IOM Equi Health project. | refugees and asylum seekers | mixed approach |
| 1. IOM,**Malta** | 2015 | Malta | to present the results of the assessment of migrant, occupational, and public health which took place in Malta within the framework of the IOM Equi Health project. | refugees and asylum seekers | mixed approach |
| 1. Jensen, N.K., et al., ***How do general practitioners experience providing care to refugees with mental health problems? A qualitative study from Denmark.*** | 2013 | Denmark | to investigate how general practitioners experience providing care to refugees with mental health problems. | refugees | qualitative |
| 1. Jensen, N.K., et al., ***Patient experienced continuity of care in the psychiatric healthcare system—a study including immigrants, refugees and ethnic Danes.*** International | 2014 | Denmark | to investigate continuity of care in the psychiatric healthcare system from the perspective of patients, including vulnerable groups such as immigrants and refugees. | refugees and immigrants | qualitative |
| 1. Joels, C., ***Impact of national policy on the health of people seeking asylum.*** | 2008 | United Kingdom | to identify when in the process asylum seekers are entitled to free NHS care. | asylum seekers | qualitative |
| 1. Johnston, V., ***Australian asylum policies: have they violated the right to health of asylum seekers?*** | 2009 | Australia | to critically examine these Australian asylum policies and assess the implications for public health practice. | asylum seekers | literature review |
| 1. Jones, C. and A.E. Williamson, ***Volunteers working to support migrants in glasgow: A qualitative study.*** | 2014 | United Kingdom | to explore the roles, motivations and experiences of volunteers who work to support asylum seekers (AS), refugees and refused asylum seekers (RAS) in Glasgow. | asylum seekers, refugees and rejected asylum seekers | qualitative |
| 1. Jonzon, R., P. et al. ***A state of limbo--in transition between two contexts: Health assessments upon arrival in Sweden as perceived by former Eritrean asylum seekers.*** | 2015 | Sweden | to explore and improve our understanding of how former asylum seekers from Eritrea perceived and experienced the health assessment during their asylum-seeking process. | Eritrean asylum seekers | qualitative |
| 1. Joshi, C., et al., ***A narrative synthesis of the impact of primary health care delivery models for refugees in resettlement countries on access, quality and coordination.*** | 2013 | NR | to identify the components of primary health care service delivery models for such populations which have been effective in improving access, quality and coordination of care. | refugees | literature review |
| 1. Kaczorowski, J.A., et al., ***Adapting clinical services to accommodate needs of refugee populations.*** | 2011 | United States | to describe our experiences with designing and adapting a variety of clinical services for youth and families with refugee status. | refugee families | qualitative |
| 1. Kaluski, D.N., et al., ***Health insurance and accessibility to health services among Roma in settlements in Belgrade, Serbia - The journey from data to policy making.*** | 2015 | Serbia | to assess the relationship between citizenship, residency and possession of health insurance cards, together with utilization of health services, among Roma residing in disadvantaged settlements in Belgrade. | Roma refugees | quantitative |
| 1. Kandasamy, T., et al., ***Obstetric risks and outcomes of refugee women at a single centre in Toronto.*** | 2014 | Canada | to determine the risk of adverse obstetric and perinatal outcomes among refugee women in Toronto. | refugee women | quantitative |
| 1. Kay, M., C. Jackson, and C. Nicholson, ***Refugee health: a new model for delivering primary health care.*** | 2010 | Australia | to describe the adaption of the Primary Care Amplification Model to enhance the delivery of health care to the refugee community. | refugees | qualitative |
| 1. Kay, M., et al., ***Understanding quality use of medicines in refugee communities in Australian primary care: a qualitative study.*** | 2016 | Australia | to identify strategies to support the quality use of medicines in refugee communities. | refugees | qualitative |
| 1. Keygnaert I, Ivanova O, Guieu A, Van Parys A-S, Leye E, K. R. ***What is the evidence on the reduction of inequalities in accessibility and quality of maternal health care delivery for migrants?*** A review of the existing evidence in the WHO European Region. 2016. | 2016 | INT | To address the following question by way of a systematic review of available academic evidence and a critical interpretive synthesis of grey literature including policy frameworks: “What is the evidence on the reduction of inequalities in accessibility and quality of maternal health care delivery for migrants? A review of the existing evidence in the WHO European Region”. | migrant women and children | mixed approach |
| 1. Keygnaert, I., et al., ***Sexual health is dead in my body: participatory assessment of sexual health determinants by refugees, asylum seekers and undocumented*** migrants in Belgium and The Netherlands. | 2014 | Belgium and The Netherlands. | to explore how refugees, asylum seekers and undocumented migrants in Belgium and The Netherlands define sexual health, search for sexual health information and perceive sexual health determinants. | refugees, asylum seekers and undocumented migrants | qualitative |
| 1. Kieft, B., et al., ***Paraprofessional counselling within asylum seekers' groups in the Netherlands: transferring an approach for a non-Western context to a*** European setting. | 2008 | The Netherlands | to increase access to basic psychosocial care to a target population that experiences difficulties in entering mental healthcare services, by a group of trained peer asylum seekers and refugees. | asylum seekers | qualitative |
| 1. Kirmayer, L.J., et al., ***Common mental health problems in immigrants and refugees: general approach in primary care.*** | 2011 | NR | to identify risk factors and strategies in the approach to mental health assessment and to prevention and treatment of common mental health problems for immigrants in primary care. | refugees and immigrants | literature review |
| 1. Klinkenberg, E., et al., ***Migrant tuberculosis screening in the EU/EEA: yield, coverage and limitations.*** | 2009 | Europe | to assess the effectiveness of tuberculosis (TB) screening methods and strategies in migrants in European Union/European Economic Area (including Switzerland) countries. | refugees, asylum seekers, immigrants | literature review |
| 1. Kouli, E., et al., ***The institutional framework regarding the rights of immigrants for access to health services in the European Union.*** | 2014 | Europe | to analyse the access rights of migrants to health services in European countries. | asylum seekers, undocumented migrants and migrants | literature review |
| 1. Kowal, S.P., C.G. Jardine, and T.M. Bubela, ***"If they tell me to get it, I'll get it. If they don't...": Immunization decision-making processes of immigrant mothers.*** | 2015 | Canada | to understand information-gathering and decision-making processes of immigrant mothers for scheduled childhood vaccines, vaccination during pregnancy, seasonal flu and pandemic vaccination. | Bhutanese, South Asian and Chinese refugee mothers | qualitative |
| 1. Kreps GLS, L. . ***Meeting the health literacy needs of immigrants populations*.** | 2008 | INT | To examine the challenges to communicating relevant information about health risks to vulnerable immigrant populations and to suggest specific communication strategies for effectively reaching and influencing these groups of people to reduce health disparities and promote public health. | immigrants | literature review |
| 1. Kurth, E., et al., ***Reproductive health care for asylum-seeking women - a challenge for health professionals.*** | 2010 | Switzerland | to identify reproductive health issues in a population of women seeking asylum in Switzerland, and to examine the care they received. | asylum seeking women | mixed approach |
| 1. Lee, HY et al. **Mental health literacy in Hmong and Cambodian elderly refugees: a barrier to understanding, recognizing, and responding to depression.** | 2010 | United States | to explore mental health literacy, specifically focusing on depression, among Southeast Asian (SEA) elderly refugees. | Hmong refugee elders | qualitative |
| 1. Lee, S.K., et al. ***Providing health information for culturally and linguistically diverse women: priorities and preferences of new migrants and refugees.*** | 2013 | Australia | to identify priority about providing health information for culturally and linguistically diverse women. | refugees and immigrant women | mixed approach |
| 1. Lee, S.K., S.C. Thompson, and D. Amorin-Woods, ***One service, many voices: enhancing consumer participation in a primary health service for multicultural women.*** Quality in Primary Care, 2009. **17**(1): p. 63-69 7p. | 2009 | Australia | to establish an active consumer reference group to assist understanding and reducing the barriers to AOD services for a heterogeneous disadvantaged group that includes individuals from different cultural, language and educational background. | refugee and immigrant women | qualitative |
| 1. Lindert, J., et al., **Mental health, health care utilisation of migrants in Europe.** | 2008 | Europe | to give an overview on (i) prevalence of mental disorders; suicide; alcohol and drug abuse; (ii) access to mental health and psychosocial care facilities of migrants in the European region, and (iii) utilisation of health and psychosocial institution of these migrants. | asylum seekers, undocumented migrants and migrants | literature review |
| 1. Ludwig, B. and H. Reed, ***When you are here, you have high blood pressure": Liberian refugees' health and access to healthcare in Staten Island, NY.*** | 2016 | United States | to examine health issues among Liberian refugees living in Staten Island and access potential barriers to accessing healthcare. | Liberian refugees | qualitative |
| 1. MacFarlane, A., et al., ***Arranging and negotiating the use of informal interpreters in general practice consultations: experiences of refugees and asylum seekers in the west of Ireland.*** | 2009 | Ireland | to compare use of professional interpreters and a trusted friend/family member. | refugees and asylum seekers | qualitative |
| 1. MacFarlane, A., et al., ***Responses to language barriers in consultations with refugees and asylum seekers: a telephone survey of Irish general practitioners.*** | 2008 | Ireland | to quantify the need for language assistance in general practice consultations and examines the experience of, and satisfaction with, methods of language assistance utilized. | refugees and asylum seekers | quantitative |
| 1. Majumder, P., et al., ***'This doctor, I not trust him, I'm not safe': the perceptions of mental health and services by unaccompanied refugee adolescents.*** | 2015 | United Kingdom | to appreciate the views and perceptions that unaccompanied minors hold about mental health and services. | unaccompanied minors | qualitative |
| 1. Manchikanti P. T**he experiences of access to primary care by afghani refugees in south east melbourne: A reflection on the public health needs of ethnic minorities.** | 2013 | Australia | to identify the acceptability of primary care and its relevance towards primary care access for Afghani refugees in south east Melbourne. | Afghan refugees | qualitative |
| 1. Mancuso, L., ***Overcoming health literacy barriers: a model for action.*** | 2011 | United States | to overcome health literacy barriers. | Indonesian asylum seekers | literature review |
| 1. Maroney, P.,et al. ***Experiences in occupational therapy with Afghan clients in Australia.*** | 2014 | Australia | to identify data and themes in literature that shed light on the utilization of health services for refugees and host population. | Afghan refugees | qualitative |
| 1. Matthews, A., et al. **How do asylum seeking and refugee women perceive and respond to preventive health care? Cervical Screening as a case study.** | 2016 | United Kingdom | to explore the facilitators and barriers to both accessing and providing cervical screening for ASR women within Glasgow. | refugees and asylum seekers | qualitative |
| 1. Matthews, A., et al. **Migration and the Media: the effect on healthcare access for asylum seekers and refugees.** | 2016 | United Kingdom | to explore how discourses in mainstream media affect asylum seeking/refugee women’s and healthcare workers ideas of deservingness for healthcare. | asylum seeker and refugee women, primary healthcare staff | qualitative |
| 1. Mayhew, M., et al., ***Facilitating refugees' access to family doctors.*** | 2015 | Canada | to describe the patient level characteristics of government-assisted refugees (GARs) who had acquired family doctors after leaving specialized refugee clinics (RC). | refugees | quantitative |
| 1. McCleary, J.S., Pet al. ***Connecting Refugees to Substance Use Treatment: A Qualitative Study.*** | 2016 | United States | to explore factors that support and prevent refugees from connecting with chemical health treatment. | social service or public health professionals who work with refugees | qualitative |
| 1. McDonald B, Gifford S, Webster K, Wiseman J, Casey S. ***Refugee resettlement in regional and rural Victoria: impacts and policy issues. Melbourne:*** Victorian Health Promotion Foundation. | 2008 | Australia | To increase understanding of the impacts of refugee regional and rural resettlement and relocation programs on the health and wellbeing of refugees; To increase understanding about the impacts of refugee regional and rural resettlement programs on regional communities; and to contribute to the development of policies and programs relevant to the resettlement of refugees in regional areas. | refugees | mixed approach |
| 1. McKeary, M. and B. Newbold, ***Barriers to care: The challenges for Canadian refugees and their health care providers.*** | 2010 | Canada | to explore the systemic barriers to health care access experienced by Canada's refugee populations. | refugees | qualitative |
| 1. McKenzie, K., ***Issues and Options for Improving Services for Diverse Populations.*** | 2015 | Canada | to outline the “Issues and Options” paper commissioned by the Mental Health Commission of Canada, which used a thorough literature review and a national consultation to develop a model for service development. | refugees, immigrants, ethnocultural, and racialized populations (IRER) | literature review |
| 1. McMichael, C. and S. Gifford, ***"It is Good to Know Now...Before it's Too Late": Promoting sexual health literacy amongst resettled young people with refugee backgrounds.*** | 2009 | Australia | to study the sexual health amongst recently arrived young people from refugee backgrounds in Melbourne, Australia. | Iraqi, Afghan, Burmese, Sudanese, Liberian, and Horn of Africa young refugees. | qualitative |
| 1. McMurray, J., et al., ***Integrated primary care improves access to healthcare for newly arrived refugees in Canada.*** | 2014 | Canada | to quantify the impact of a partnership between a dedicated health clinic for government assisted refugees (GARs), a local reception centre and community providers, on wait times and referrals. | refugees | quantitative |
| 1. Médecins Sans Frontières, *N****OT CRIMINALS. Médecins Sans Frontières exposes conditions for undocument ed migrants an d as ylum seekers in Maltese* detention centres** | 2009 | Malta | to describe the provision of health careby Médecins Sans Frontières (MSF) started providing health care in Maltese detention centres for undocumented migrants and asylum seekers. | asylum seekers and undocumented migrants | quantitative |
| 1. Mei Lan, F., et al., ***Experiencing 'pathologized presence and normalized absence'; understanding health related experiences and access to health care among Iraqi and Somali asylum seekers, refugees and persons without legal status.*** | 2015 | United Kingdom | to explore health and health care experiences of Somali and Iraqi asylum seekers, refugees and persons without legal status, highlighting 'minoritization' processes and the 'pathologization' of difference as analytical lenses to understand the multiple layers of oppression that contribute to health inequities. | asylum seekers, undocumented migrants and refugees. | qualitative |
| 1. Merry, L.A., et al., ***Refugee claimant women and barriers to health and social services post-birth.*** | 2011 | Canada | to gain greater understanding of the barriers these vulnerable migrant women face in accessing health and social services postpartum. | asylum seeking women | qualitative |
| 1. Milosevic, D., I.H. Cheng, and M.M. Smith, ***The NSW refugee health service: Improving refugee access to primary care.*** | 2012 | Australia | to describe the area of need, the innovative strategies that have been developed by specific organisations to address this need, and make recommendations to help GPs improve access to disadvantaged populations in their own communities. | refugees | qualitative |
| 1. Mirdal, G.M., et al. ***Traumatized refugees, their therapists, and their interpreters: three perspectives on psychological treatment.*** | 2012 | United States | to study how traumatized refugees, their therapists, and their interpreters perceive both curative and hindering factors in psychological therapy, thereby highlighting the mediators of change in a transcultural clinical setting. | refugees, therapists and interpreters | quantitative |
| 1. Mirza, M. and A.W. Heinemann, ***Service needs and service gaps among refugees with disabilities resettled in the United States.*** | 2012 | United States | to examine the adequacy of existing service systems in addressing the needs of refugees with disabilities resettled in the U.S.A. | disabled refugees | qualitative |
| 1. Mirza, M., et al., ***Barriers to Healthcare Access Among Refugees with Disabilities and Chronic Health Conditions Resettled in the US Midwest.*** | 2014 | United States | to explore the access to appropriate healthcare services of disabled refugees in order to identify service disparities and improve interventions. | disabled and chronically ill refugees | qualitative |
| 1. Mitschke, DB, et al. **Uncovering Health and Wellness Needs of Recently Resettled Karen Refugees from Burma.** | 2011 | United States | to identify obstacles to acculturation long after initial resettlement of refugees. | Karen refugees | qualitative |
| 1. Morris, M.D., et al., ***Healthcare barriers of refugees post-resettlement.*** | 2009 | United States | to identify the health needs beyond a health assessment completed upon entry. | refugees | qualitative |
| 1. Mucic, D., ***Transcultural telepsychiatry and its impact on patient satisfaction.*** | 2010 | Denmark | to improve access to culturally appropriate care providers (i.e. culturally competent, bilingual clinicians) by the use of videoconferencing. | asylum seekers, refugees and migrants | quantitative |
| 1. Murray, L., et al., ***The experiences of African women giving birth in Brisbane, Australia.*** | 2010 | Australia | to uncover first-person descriptions of the birth experiences of African refugee women in Brisbane, Australia, and to explore the common themes that emerged from their experiences. | African refugee women | qualitative |
| 1. Museru, O.I., et al., ***Hepatitis B virus infection among refugees resettled in the U.S.: high prevalence and challenges in access to health care.*** | 2010 | United States | to assess the epidemiology of HBV and entry into medical care in refugee communities resettled in the State of Georgia over a five-year period: 2003-2007. | refugees | quantitative |
| 1. Nazzal, K.H., et al., ***An innovative community-oriented approach to prevention and early intervention with refugees in the United States.*** | 2014 | United States | to present a community-oriented prevention and early intervention model that can be used with newly arrived refugees with limited English proficiency. | refugees | qualitative |
| 1. Newbold, K.B., et al. ***Access to Health Care: The Experiences of Refugee and Refugee Claimant Women in Hamilton, Ontario.*** | 2013 | Canada | to explore the accessibility of health services from the viewpoints of service providers, and refugee and refugee claimant women in Hamilton, Ontario. | refugee and aslum-seeking women | qualitative |
| 1. Nicol, P., et al., ***Informing a culturally appropriate approach to oral health and dental care for pre-school refugee children: a community participatory study.*** | 2014 | Australia | to provide a deeper understanding of the refugee experience related to early oral health by exploring pre-school refugee families (i) understanding of ECC and child oral health, (ii) experiences of accessing dental services and (iii) barriers and enablers for achieving improved oral health. | children in families of recently settled refugees | qualitative |
| 1. Njeru, J.W., et al., ***Stories for change: development of a diabetes digital storytelling intervention for refugees and immigrants to Minnesota using qualitative methods.*** | 2015 | United States | to develop a diabetes digital storytelling intervention with and for immigrant and refugee populations. | Somali and Latino immigrants and refugees | qualitative |
| 1. Nkulu Kalengayi, F K. **Perspectives of asylum seekers and refugees on health assessment:"It is a requirement that benefits everyone"** | 2014 | Sweden | to explore asylum ‘seekers perceptions and experiences of health assessment. | asylum seekers | qualitative |
| 1. Norredam, M. **Migration and health: Organising access to EU health care systems for migrants.** | 2016 | Europe | to describe the formal and informal barriers related to access and to suggest solutions. | refugees and asylum seekers | qualitative |
| 1. Norredam, M., ***Migrants' access to healthcare.*** | 2011 | Denmark | to increase the understanding of migrants' access to healthcare by exploring two study aims: 1) Are there differences in migrants' access to healthcare compared to that of non-migrants? (substudy I and II); and 2) Why are there possible differences in migrants' access to healthcare compared to that of non-migrants? (substudy III and IV). | asylum seekers, undocumented migrants, refugees and migrants | quantitative |
| 1. O'Donnell, C.A., et al., ***Asylum seekers' expectations of and trust in general practice: a qualitative study.*** | 2008 | United Kingdom | to explore how migrants' previous knowledge and experience of health care influences their current expectations of health care in a system relying on clinical generalists performing a gatekeeping role. | asylum seekers | qualitative |
| 1. O'Mahony, J. and T. Donnelly, ***Immigrant and refugee women's post-partum depression help-seeking experiences and access to care: a review and analysis of the literature.*** | 2010 | NR | to analyse the literature about post-partum depression and the positive and negative factors, which may influence immigrant and refugee women's health seeking behaviour and decision making about post-partum care. | refugee and immigrant women | literature review |
| 1. O'Mahony, J.M. and T.T. Donnelly, ***How does gender influence immigrant and refugee women's postpartum depression help-seeking experiences?*** | 2013 | Canada | to explore how cultural, social, political, historical and economic factors intersect with race, gender and class to influence the ways in which immigrant and refugee women seek help to manage post-partum depression. | refugee and immigrant women | qualitative |
| 1. O'Mara, B., ***Social media, digital video and health promotion in a culturally and linguistically diverse Australia.*** | 2013 | Australia | to identify opportunities and challenges when using social media with communities from diverse cultural and linguistic backgrounds. | refugees, migrants and communities from diverse cultural and linguistic backgrounds | literature review |
| 1. O’Reilly-de Brún, et al. **Involving migrants in the development of guidelines for communication in cross-cultural general practice consultations: a participatory learning and action research project**. | 2016 | Ireland | to involve migrants and other key stakeholders in a participatory dialogue to develop a guideline for enhancing communication in cross-cultural general practice consultations. | refugees and immigrants | qualitative |
| 1. Odunukan, O.W., et al., ***Provider and interpreter preferences among Somali women in a primary care setting.*** | 2015 | United States | to elucidate provider and interpreter preferences during clinical encounters according to gender and race among Somali women in the United States. | Somali refugee women | qualitative |
| 1. Oktem, P., et al. **Migrant women's access to healthcare in Turkey.** | 2016 | Turkey | to address migrant and refugee women’s access to healthcare in Turkey, which remained an under-researched topic, from a gender and human rights perspective. | refugees and immigrant women and key informants | qualitative |
| 1. Okunseri, C., et al., ***Hmong adults self-rated oral health: a pilot study.*** | 2008 | United States | to describe the self-related oral health, self-rated general health, and use of dental/physician services; and to identify the factors associated with self-related oral health among Hmong adults. | Hmong refugees | quantitative |
| 1. Percac-Lima, S., et al., ***Decreasing disparities in breast cancer screening in refugee women using culturally tailored patient navigation.*** | 2013 | United States | to evaluate whether a PN program for refugee women decreases disparities in breast cancer screening. | Somali, Arabic, or Serbo-Croatian (Bosnian) refugee women | qualitative |
| 1. Percac-Lima, S., et al., ***Patient navigation to improve breast cancer screening in Bosnian refugees and immigrants.*** | 2012 | United States | to report the outcomes of a breast cancer screening patient navigation program for refuge/immigrant women from Bosnia. | Bosnian refugee/immigrant women | quantitative |
| 1. Pieper, H.O., et al. ***The impact of direct provision accommodation for asylum seekers on organisation and delivery of local primary care and social care services: A case study.*** | 2011 | Ireland | to explore he impact of direct provision accommodation on organisation and delivery of local primary care and social care services in the community. | stakeholders | qualitative |
| 1. Pimentel, VM & Eckardt, MJ. **More than interpreters needed: the specialized care of the immigrant pregnant patient.** | 2014 | NR | to provide an overview of the challenges and interventions to maximize health outcomes for the immigrant pregnant woman. | Immigrant pregnant women | qualitative |
| 1. Piwowarczyk, L., et al., ***Congolese and Somali beliefs about mental health services.*** | 2014 | United States | to examine both concepts of mental illness in addition to attitudes and beliefs about treatment as well as potential barriers to accessing mental health services. | Congolese and Somali men and women | qualitative |
| 1. Platform for International Cooperation on Undocumented Migrants. ***Undocumented Children in Europe: Invisible Victims of Immigration Restrictions***. | 2008 | Europe | to investigate the particular vulnerability that characterizes these children and analyse their specific needs and problems in various European countries. | asylum-seeking, refugee and migrant children | mixed approach |
| 1. Posselt, M., et al., ***Merging perspectives: obstacles to recovery for youth from refugee backgrounds with comorbidity.*** | 2015 | Australia | to identify challenges encountered by young people from refugee backgrounds with co-existing mental health (MH) and alcohol and other drug (AOD) problems (comorbidity) and sought to compare the perspectives of refugee youth and service providers in a metropolitan region of Adelaide, South Australia. | African, Afghan, Bhutanese, workers from MH, AOD and refugee support services | qualitative |
| 1. Pottie, K., et al., ***Improving delivery of primary care for vulnerable migrants: Delphi consensus to prioritize innovative practice strategies.*** | 2014 | Canada | to identify and prioritize innovative strategies to address the health concerns of vulnerable migrant populations. | primary care practitioners, including family physicians and nurse practitioners | qualitative |
| 1. Poureslami, I., et al., ***Bridging immigrants and refugees with early childhood development services: partnership research in the development of an effective service model.*** . | 2013 | Canada | to assess the different meanings, understandings, and practices relating to early childhood development services, examine the ways in which behavioural, cultural, and institutional practices may influence early childhood development services access and use of services; and contribute to the development of a culturally competent definition, measure, and model for early childhood development services that is applicable to ethno-cultural communities. | Chinese, Korean, Iranian and Afghani refugees and immigrants, ECD service providers, community educators, and facilitators | qualitative |
| 1. Power D & Pratt R. ***Karen refugees from Burma: focus group analysis*** | 2012 | United States | to describe the health experiences of a recently arrived group of refugees, the Karen from Burma, in an American midwestern city. | Karen refugees | qualitative |
| 1. Priebe, S., et al., ***Good practice in mental health care for socially marginalised groups in Europe: A qualitative study of expert views in 14 countries.*** | 2012 | Europe | to explore the experiences and views of experts in 14 European countries regarding mental health care for six socially marginalised groups: long-term unemployed; street sex workers; homeless; refugees/asylum seekers; irregular migrants and members of the travelling communities. | refugees, asylum seekers, irregular migrants, long-term unemployed, street sex workers, homeless, and members of the travelling communities. | qualitative |
| 1. Qayyum, M.A., et al., ***The provision and sharing of information between service providers and settling refugees.*** | 2014 | Australia | to understand the provision and sharing of information between service providers and settling refugees while refugees transit to new living environments. | refugees and service providers from community and public sector organizations | qualitative |
| 1. Rabiee, F., Smith, P. **Equity in Mental Health Service Provision for African Caribbean, Black African Refugees and Asylum Seekers.** | 2016 | United Kingdom | to examine understanding of mental health nd experience of accessing mental health services from the perspectives of black African and African Caribbean mental health service users and their carers. | African Caribbean, Black African refugees and asylum seekers | qualitative |
| 1. Ratnam, S., et al. **The "migrant kit": a new guide for migrant-friendly care in a Swiss paediatric hospital** | 2016 | Switzerland | to assess the guidelines (the "migrant kit") for residents and all staff in outpatient and inpatient units in a Swiss hospital. | health care professionals | quantitative |
| 1. Razavi, M.F., et al., ***Experiences of the Swedish healthcare system: An interview study with refugees in need of long-term health care.*** Scandinavian Journal of Public Health, 2011. **39**(3): p. 319-325. | 2011 | Sweden | to examine the viewpoints of nine refugees in a county in Sweden, with a known chronic disease or functional impairment requiring long-term medical care, on their contacts with care providers regarding treatment and personal needs. | refugees | qualitative |
| 1. Reavy, K., et al., ***A new clinic model for refugee health care: adaptation of cultural safety.*** | 2012 | United States | to differentiate the role of C.A.R.E. Clinic Health Advisor from certified medical interpreter and to evaluate the lived experiences of each role. | refugees | qualitative |
| 1. Rechel, B., et al. **Health system responses to the influx of refugees in Europe.** | 2016 | Europe | to present the results of a research project by the European Observatory on Health Systems and Policies and the World Health Organization Regional Office for Europe. | refugees | qualitative |
| 1. Redwood-Campbell, L., et al., **Understanding the health of refugee women in host countries: lessons from the Kosovar re-settlement in Canada. Prehosp Disaster Med, 2008.** 23(4): p. 322-7. | 2008 | Canada | to describe the results of a self-administered survey regarding women's health issues and experiences with health services after the arrival of refugees and the sponsor group's experience related to women's health care. | refugee women | quantitative |
| 1. Reichlin, R., et al. **Applying a Community-Based Participatory Research Approach to Improve Asylum-Seekers’ Access to Healthcare in Israel.** | 2016 | Israel | to advocate inclusion in Israel's public healthcare system, and b) to address root causes of health inequities through facilitating participation of the asylum-seeking communities in political decision-making processes. | Eritrean asylum seekers, local activists and academics | qualitative |
| 1. Rew, K.T., et al., ***Immigrant and refugee health: cross-cultural communication.*** | 2014 | United States | to provide guidance for cross cultural communication. | refugees and immigrants | literature review |
| 1. Reynolds, B. and J. White, ***Seeking asylum and motherhood: health and wellbeing needs.*** | 2010 | United Kingdom | to investigate the health and wellbeing needs of pregnant asylum-seeking women and those with young babies living in initial accommodation centres. | pregnant asylum-seeking women, asylum seeking mothers | qualitative |
| 1. Riggs, E., et al., ***'We are all scared for the baby': promoting access to dental services for refugee background women during pregnancy.*** | 2016 | Australia | to describe Afghan and Sri Lankan women's knowledge and beliefs surrounding maternal oral health, barriers to accessing dental care during pregnancy, and to present the perspectives of maternity and dental service providers in relation to dental care for pregnant women. | Afghan & Sri Lankan refugees, dental staff including clinicians and administrative staff, and midwives. | qualitative |
| 1. Riggs, E., et al., ***Accessing maternal and child health services in Melbourne, Australia: reflections from refugee families and service providers.*** | 2012 | Australia | to explore experiences of using maternal and child health services, from the perspective of families from refugee backgrounds and service providers. | Karen, Iraqi, Assyrian Chaldean, Lebanese, South Sudanese and Bhutanese refugees women, MCH nurses, other healthcare providers and bicultural workers. | qualitative |
| 1. Robinson, K., ***Supervision Found Wanting: Experiences of Health and Social Workers in Non-Government Organisations Working with Refugees and Asylum Seekers.*** | 2013 | Australia/UK | to explores the role and function of supervision in social work with refugees and asylum seekers. | health and social workers | qualitative |
| 1. Ross, L., et al. **Improving the management and care of refugees in Australian hospitals: a descriptive study.** | 2016 | Australia | to investigate healthcare provider perceptions of the impact of refugee patients at two public hospitals, one rural and one urban, in designated refugee resettlement areas. | refugees | quantitative |
| 1. Rousseau, C. and J. Guzder, ***School-based prevention programs for refugee children.*** Child Adolesc Psychiatr Clin N Am, 2008. **17**(3): p. 533-49, viii. | 2008 | NR | to review existing school-based prevention programs. | refugee children | literature review |
| 1. Russo, A., et al., ***A qualitative exploration of the emotional wellbeing and support needs of new mothers from Afghanistan living in Melbourne, Australia.*** BMC Pregnancy Childbirth, 2015. **15**: p. 197. | 2015 | Australia | to explored the experiences of Afghan women living in Melbourne throughout pregnancy, birth, and early motherhood, and gain insight into the aspects of their experiences that they perceive as positively and negatively impacting their emotional wellbeing. | Afghan refugee women | qualitative |
| 1. Saadi, A., B. Bond, and S. Percac-Lima, ***Perspectives on preventive health care and barriers to breast cancer screening among Iraqi women refugees.*** J Immigr Minor Health, 2012. **14**(4): p. 633-9. | 2012 | United States | to assess the perspectives of Iraqi women refugees on preventive care and perceived barriers to breast cancer screening. | Iraqi refugee women | qualitative |
| 1. Sandahl, H., et al., ***Policies of access to healthcare services for accompanied asylum-seeking children in the Nordic countries.*** Scand J Public Health, 2013. **41**(6): p. 630-6. | 2013 | Nordic countries | to compare policies of access to healthcare services, including physical examination and screening for mental health problems on arrival, for accompanied asylum-seeking children in the Nordic countries. | asylum seeking children | literature review |
| 1. Sandhu, S., et al., ***Experiences with treating immigrants: a qualitative study in mental health services across 16 European countries.*** Soc Psychiatry Psychiatr Epidemiol, 2013. **48**(1): p. 105-16. | 2013 | Europe | to explore professionals' experiences of delivering care to immigrants in districts densely populated with immigrants across Europe. | asylum seekers, undocumented migrants, refugees and migrants | qualitative |
| 1. Sandikli, B. et al. **Role of NGOs in addressing the needs of Syrian refugees living in Istanbul** | 2016 | Turkey | to describe the role of NGOs in supporting migrants to access health care services. | Syrian refugee women, representatives of Syrian and Turkish NGOs, doctors and decision makers | qualitative |
| 1. Schulz, T.R., et al., ***Improvements in patient care: videoconferencing to improve access to interpreters during clinical consultations for refugee and immigrant patients*** | 2015 | Australia | to demonstrate the suitability of accessing interpreters via videoconference for medical consultations and to assess doctor and patient perceptions of this compared with either on-site or telephone interpreting. | refugee and immigrants | quantitative |
| 1. Schulz, T.R., et al., ***Telehealth: experience of the first 120 consultations delivered from a new refugee telehealth clinic.*** Intern Med J, 2014. **44**(10): p. 981-5. | 2014 | Australia | to assess the demographic and disease profile of refugee patients attending a new tele-health clinic, to calculate the patient travel avoided, to examine technical challenges and assessed the performance of two videoconferencing solutions using different bandwidth and latencies. | refugees | quantitative |
| 1. Scott, P., ***Black African asylum seekers' experiences of health care access in an eastern German state.*** International Journal of Migration, Health and Social Care, 2014. **10**(3): p. 134-147. | 2014 | Germany | to examine how access to health care for (rejected) asylum seekers in an eastern German state is structured and experienced and to consider the implications for their human rights. | asylum seekers and rejected asylum seekers | qualitative |
| 1. Seery, T., H. Boswell, and A. Lara, ***Caring for refugee children.*** Pediatrics in Review, 2015. **36**(8): p. 323-338. | 2015 | United States | to provide guidance to care for refugee children. | refugee children | literature review |
| 1. Segala, D., et al. **Health education and HIV test offer in a population of refugees and asylum seekers: an experience in Ferrara area.** | 2016 | Italy | to improve HIV/AIDS-related knowledge within migrants, refugees and asylum seekers, to favour access to public health service and HIV/STDs test. | refugees and asylum seekers | quantitative |
| 1. Sethi, B., ***Service delivery on rusty health care wheels: implications for visible minority women.*** J Evid Based Soc Work, 2013. **10**(5): p. 522-32. | 2013 | Canada | to demonstrate how immigrant/refugee women's access to health services is influenced by both immigration and health policies. | refugee and immigrant women | literature review |
| 1. Sheikh, M. and C.R. MacIntyre, ***The impact of intensive health promotion to a targeted refugee population on utilisation of a new refugee paediatric clinic at the children's hospital at Westmead.*** Ethn Health, 2009. **14**(4): p. 393-405. | 2009 | Australia | to evaluate the impact of intensive promotion of a new health service to a targeted refugee population, recently resettled in Sydney, and the role of early social connection and membership of social group in promoting health service utilisation of refugees. | refugees | quantitative |
| 1. Sheikh, M., et al., ***Equity and access: understanding emergency health service use by newly arrived refugees.*** Med J Aust, 2011. **195**(2): p. 74-6. | 2011 | Australia | to determine issues that affect newly resettled refugees in accessing an emergency department (ED). | Middle East and Africa refugees | quantitative |
| 1. Show JS, et al. ***The role of culture in health literacy and chronic disease screening and management.*** | 2009 | INT | to examine cultural influences on health literacy, cancer screening and chronic disease outcomes. | asylum-seekers, refugees and migrants | literature review |
| 1. Simich L. ***Health literacy, immigrants and mental health.*** | 2010 | Canada | to defines health literacy and its implications for immigrants in Canada. | refugees and migrants | literature review |
| 1. Simonnot, N., et al. **Health and access to care for migrants facing multiple vulnerabilities in Europe.** | 2016 | INT | to collect data on health care and health care access for asylum seekers in Europe . | asylum seekers, undocumented migrants and migrants | quantitative |
| 1. Sinha, S., S. Uppal, and A. Pryce, ***'I had to cry': exploring sexual health with young separated asylum seekers in East London.*** Diversity in Health & Social Care, 2008. **5**(2): p. 101-111 11p. | 2008 | United Kingdom | to explore sexual health and sexual exploitation for those young asylum seekers separated from parents. | unaccompanied minors | qualitative |
| 1. Spike, E.A., M.M. Smith, and M.F. Harris, ***Access to primary health care services by community-based asylum seekers.*** Med J Aust, 2011. **195**(4): p. 188-91. | 2011 | Australia | to determine whether community-based asylum seekers experience difficulty in gaining access to primary health care services, and to determine the impact of any difficulties described. | asylum seekers, health care practitioners and staff | qualitative |
| 1. Sudbury, H. and A. Robinson, **Barriers to sexual and reproductive health care for refugee and asylum-seeking women.** British Journal of Midwifery, 2016. **24**(4): p. 275-281. | 2016 | United Kingdom | to examine barriers to sexual and reproductive health care for refugee and asylum-seeking women, exploring how issues can be addressed and ameliorated by midwives and the wider health-care team during pregnancy. | refugee and asylum-seeking women | literature review |
| 1. Sullivan, C.H., ***Partnering with community agencies to provide nursing students with cultural awareness experiences and refugee health promotion access.*** J Nurs Educ, 2009. **48**(9): p. 519-22. | 2009 | United States | to describe a teaching-learning strategy emphasizing the community partnership between a baccalaureate school of nursing, an immigrant-refugee program, and a community literacy program in a rural state. | refugees | literature review |
| 1. Swe, H.M. and M.W. Ross, ***Refugees from Myanmar and their health care needs in the US: A qualitative study at a refugee resettlement agency.*** International Journal of Migration, Health and Social Care, 2010. **6**(1): p. 15-25. | 2010 | United States | to look at the refugees' perspectives and identified the gaps in their understanding of the US health care system, health-seeking behaviours and challenges in using health care in the United States. | Myanmar refugees | quantitative |
| 1. Szajna, A. and J. Ward, ***Access to health care by refugees: a dimensional analysis.*** Nurs Forum, 2015. **50**(2): p. 83-9. | 2015 | United States | to analyse access to healthcare services by the refugee population. | refugees | qualitative |
| 1. Tastsoglou, E., et al., ***(En) gendering vulnerability: Immigrant service providers' perceptions of needs, policies, and practices related to gender and women refugee claimants in Atlantic Canada.*** Refuge, 2014. **30**(2): p. 67-78. | 2014 | Canada | to describe the experiences and perceptions of immigrant service providers in relation to gender and women refugee claimants. | refugee women | qualitative |
| 1. Taylor, K., ***Asylum seekers, refugees, and the politics of access to health care: a UK perspective.*** | 2009 | United Kingdom | to considers the wider ethical, moral, and political issues that may arise from the politics of access to health care. | asylum seekers | literature review |
| 1. The World Health Organization. ***HEALTH OF MIGRANTS − THE WAY FORWARD***. Report of a global consultation. | 2010 | INT | This report includes a summary of the Global Consultation based on keynote addresses, presentations and debates, as well as a summary of the recommendations on future priorities and actions. It concludes with an outline for an operational framework based on the inputs from the consultation participants, and a “way forward” as formulated by the Organizers. | asylum-seekers, refugees and migrants | qualitative |
| 1. Thomson, M.S., et al., ***Improving Immigrant Populations’ Access to Mental Health Services in Canada: A Review of Barriers and Recommendations.*** Journal of Immigrant and Minority Health, 2015. **17**(6): p. 1895-1905. | 2015 | Canada | to review the relevant literature on immigrants’ access to mental health services in Canada. | refugees and immigrants | literature review |
| 1. Tobin, C., J. Murphy-Lawless, and C.T. Beck, ***Childbirth in exile: asylum seeking women's experience of childbirth in Ireland.*** Midwifery, 2014. **30**(7): p. 831-8. | 2014 | Ireland | to gain insight into women's experiences of childbirth in Ireland while in the process of seeking asylum. | asylum seeking pregnant women | qualitative |
| 1. Torres, S., et al., ***Improving health equity: The promising role of community health workers in Canada.*** Healthcare Policy, 2014. **10**(1): p. 73-85. | 2014 | Canada | to explore the challenges, successes and unrealized potential of community health workers in facilitating culturally responsive access to healthcare and other social services for new immigrants and refugees. | refugees and immigrants | qualitative |
| 1. Torun, P., et al. **A health and health care needs assessment for the Syrian community living in Zeytinburnu district of Istanbul** | 2016 | Turkey | to assess the needs of urban refugees. | Syrian refugees | mixed approach |
| 1. UNHCR, **Regional refugee and migrant response plan for Europe. Eastern Mediterranean and Wester Balkans route.** | 2016 | Turkey, Greece, Macedonia, Serbia, Croatia, Slovenia | to present a set of measures that will enable the humanitarian community to contribute to the protection of refugees and vulnerable migrants, as well as the human rights of all people involved. | refugees and asylum seekers | qualitative |
| 1. United Nations High Commissioner for Refugees. ***Ensuring Access to Health Care: Operational Guidance on Refugee Protection and Solutions in Urban Areas.*** | 2011 | INT | To provide guidance for UNHCR country programmes to advocate for and facilitate access to (and when necessary provide and/or support) quality public health services for refugees equivalent to those available to the national population. | urban refugees | qualitative |
| 1. United Nations High Commissioner for Refugees. ***Study of the Office of the United Nations High Commissioner for Human Rights on challenges and best practices in the implementation of the international framework for the protection of the rights of the child in the context of migration.*** | 2010 | INT | To set out the specific standards and principles that informs the international framework of protection of the rights of the child in the context of migration. | asylum-seekers, refugees and migrants | literature review |
| 1. Ussher, J.M., et al., ***Purity, Privacy and Procreation: Constructions and Experiences of Sexual and Reproductive Health in Assyrian and Karen Women Living in Australia.*** Sexuality and Culture, 2012. **16**(4): p. 467-485. | 2012 | Australia | to examine the constructions and experiences of reproductive and sexual health, and associated services, in Assyrian and Karen women who had arrived in Australia as refugees. | Assyrian and Karen refugee women | qualitative |
| 1. Vanthuyne, K., et al., ***Health workers' perceptions of access to care for children and pregnant women with precarious immigration status: Health as a right or a privilege?*** Social Science & Medicine, 2013. **93**: p. 78-85 8p. | 2013 | Canada | to explore the consequences of the cuts to healthcare coverage for refugee claimants, focusing on the perceptions of healthcare workers. | pregnant women and children asylum seekers | quantitative |
| 1. Vermette, D., et al., ***Healthcare Access for Iraqi Refugee Children in Texas: Persistent Barriers, Potential Solutions, and Policy Implications.*** Journal of Immigrant & Minority Health, 2015. **17**(5): p. 1526-1536 11p. | 2015 | United States | to identify access barriers to healthcare and potential interventions to improve access for Iraqi refugee children. | Iraqi refugee children | qualitative |
| 1. Wagner, J., et al., ***Diabetes among refugee populations: what newly arriving refugees can learn from resettled Cambodians.*** Curr Diab Rep, 2015. **15**(8): p. 56. | 2015 | NR | to reviews rates of cardio metabolic disease and type 2 diabetes among refugees and highlights their unique risk factors including history of malnutrition, psychiatric disorders, psychiatric medications, lifestyle changes toward urbanization and industrialization, social isolation, and a poor profile on the social determinants of health. | refugees | literature review |
| 1. Wahoush, E.O., ***Equitable health-care access: the experiences of refugee and refugee claimant mothers with an ill preschooler.*** Can J Nurs Res, 2009. **41**(3): p. 186-206. | 2009 | Canada | to explore the access to health services for preschool children in refugee or refugee claimant families living in Canada. | refugee and asylum-seeking children | mixed approach |
| 1. Wangdahl, J., et al., ***Health literacy and refugees' experiences of the health examination for asylum seekers - a Swedish cross-sectional study.*** BMC Public Health, 2015. **15**: p. 1162. | 2015 | Sweden | to investigate refugees' experiences of communication during their health examination for asylum seekers and the usefulness of that examination, and whether health literacy is associated with those experiences. | asylum seekers | quantitative |
| 1. Weine, S., et al., ***Evaluating a multiple-family group access intervention for refugees with PTSD.*** J Marital Fam Ther, 2008. **34**(2): p. 149-64. | 2008 | United States | to analyse the effects of a multiple-family group in increasing access to mental health services for refugees with posttraumatic stress disorder (PTSD). | Bosnian refugee families | quantitative |
| 1. Wohler, Y. & Dantas, JA. **Barriers Accessing Mental Health Services Among Culturally and Linguistically Diverse (CALD) Immigrant Women in Australia: Policy Implications.** | 2016 | NR | to describe barriers that immigrant and refugee women from diverse ethnic backgrounds encounter in accessing mental healthcare in various settings. | refugee and immigrant women | literature review |
| 1. Wojnar, D.M., ***Perinatal Experiences of Somali Couples in the United States.*** JOGNN: Journal of Obstetric, Gynecologic & Neonatal Nursing, 2015. **44**(3): p. 358-369 12p. | 2015 | United States | to explore the perspectives of Somali couples on care and support received during the perinatal period in the United States. | Somali refugees | qualitative |
| 1. Wollersheim, D., et al., ***Constant connections: piloting a mobile phone-based peer support program for Nuer (southern Sudanese) women.*** Aust J Prim Health, 2013. **19**(1): p. 7-13. | 2013 | Australia | to find out how to use mobile phone-based peer support to improve the psychosocial health of, and facilitate settlement in a group of Nuer refugee women. | refugee women | qualitative |
| 1. Wollscheid, S., et al. **Effect of Interventions to Facilitate Communication Between Families or Single Young People with Minority Language Background and Public Services: A Systematic Review.** | 2015 | INT | to examine whether interventions to facilitate communication between public services, on the one hand, and minority language children and youth or families with an immigrant background, on the other, are effective. | children, young people and families with minority-language and immigrant backgrounds | literature review |
| 1. Woodland ,L., et al**.Evaluation of a school screening programme for young people from refugee backgrounds.** | 2016 | Australia | To describe the development of the Optimising Health and Learning Program, guided by the only available published framework for the delivery of health services to newly arrived refugee children and report on the evaluation of the programme. | youth refugees | mixed approach |
| 1. Woodland, L., et al., ***Health service delivery for newly arrived refugee children: a framework for good practice.*** J Paediatr Child Health, 2010. **46**(10): p. 560-7. | 2010 | Australia | to propose a framework for good practice to promote improved access, equity and quality of care in service delivery for newly arrived refugee children. | refugee children | literature review |
| 1. Xiao, L.D., et al. ***Perceived Challenges in Dementia Care by Vietnamese Family Caregivers and Care Workers in South Australia.*** | 2015 | Australia | to explore the perceived challenges of dementia care from Vietnamese family caregivers and Vietnamese care workers. | Vietnamese refugee families, Vietnamese care workers | qualitative |
| 1. Yelland, J., et al., ***Compromised communication: A qualitative study exploring Afghan families and health professionals' experience of interpreting support in Australian maternity care.*** BMJ Quality and Safety, 2016. **25**(4): p. e1. | 2016 | Australia | to explore Afghan women and men's experience of language support during pregnancy, labour and birth, and health professionals' experiences of communicating with clients of refugee background with low English proficiency. | Afghan refugees | qualitative |
| 1. Yelland, J., et al., ***Maternity services are not meeting the needs of immigrant women of non-English speaking background: Results of two consecutive Australian population based studies.*** Midwifery, 2015. **31**(7): p. 664-670. | 2015 | Australia | to compare the views and experiences of immigrant women of non-English speaking background (NESB) giving birth in Victoria, Australia with those of women who were born in Australia. | refugee and immigrant women | quantitative |
| 1. Yun, K., et al., ***Help-Seeking Behavior and Health Care Navigation by Bhutanese Refugees.*** Journal of Community Health, 2016. **41**(3): p. 526-534. | 2016 | United States | to document barriers to care, help-seeking behaviours, and the impact of a community-based patient navigation intervention on patient activation levels among Bhutanese refugees in the U.S. | Bhutanese refugees | quantitative |
