# Supplementary material for: Supporting access to healthcare for refugees and migrants in European countries under particular migratory pressure
Source: BMC Health Serv Res. 2019 Jul 23;19:513. doi: 10.1186/s12913-019-4353-1 (PMC6651950; doi:10.1186/s12913-019-4353-1)
Supplement: Supplementary file 3 — List of references included in the systematic review divided by barriers. (DOCX 58 kb) [file 12913_2019_4353_MOESM3_ESM.docx]

Supplementary file 3: list of complete references included in the systematic review divided by type of barriers/solutions
*(Please note that a same study could focus on more than one barrier or solution)*

**Included studies on barriers and solutions related to accessing healthcare services in general for refugees and migrants**

1. Asgary, R. and N. Segar, *Barriers to health care access among refugee asylum seekers.* J Health Care Poor Underserved, 2011. **22**(2): p. 506-22.
2. Aspinall, P., *Vulnerable Migrants, Gypsies and Travelers, People Who Are Homeless, and Sex Workers: A Review and Synthesis of Interventions/Service Models that Improve Access to Primary Care & Reduce Risk of Avoidable Admission to Hospital*, in *Inclusive Practice*. 2014, University of Kent: Kent, United Kingdom.
3. Baird, M.B., *Well-being in refugee women experiencing cultural transition.* ANS Adv Nurs Sci, 2012. **35**(3): p. 249-63.
4. Bellamy, K., et al., *Access to medication and pharmacy services for resettled refugees: a systematic review.* Aust J Prim Health, 2015. **21**(3): p. 273-8.
5. Beltran-Avery, P.P., *'The role of the health care interpreter’, National Council on Interpreting in Health Care. 2011. Online. Available at: www.ncihc.org/workingpapers.htm.*
6. Bischoff, A. and K. Denhaerynck, *What do language barriers cost? An exploratory study among asylum seekers in Switzerland.* BMC Health Serv Res, 2010. **10**: p. 248.
7. Bischoff, A., P. Hudelson, and P.A. Bovier, *Doctor-patient gender concordance and patient satisfaction in interpreter-mediated consultations: an exploratory study.* J Travel Med, 2008. **15**(1): p. 1-5.
8. Bodenmann, P. and A.R. Green, *Health disparities: Local realities and future challenges.* Revue Medicale Suisse, 2012. **8**(364): p. 2282-2286.
9. Bogenschutz, M., *"We find a way": challenges and facilitators for health care access among immigrants and refugees with intellectual and developmental disabilities.* Med Care, 2014. **52**(10 Suppl 3): p. S64-70.
10. Boise, L., et al., *African refugee and immigrant health needs: report from a community-based house meeting project.* Prog Community Health Partnersh, 2013. **7**(4): p. 369-78.
11. Bradby, H., et al., *Public health aspects of migrant health: a review of the evidence on health status for refugees and asylum seekers in the European Region*, in *Health Evidence Network synthesis report*. 2015.
12. Bradby, H., et al. *Refugees and asylum seekers in the European Region - reviewing the research evidence* in *6th European Conference on Migrant and Ethnic Minority Health*. 2016. Oslo, Norway. https://www.duo.uio.no/bitstream/handle/10852/55205/abstract-book-eupha2016.pdf?sequence=3&isAllowed=y.
13. Brolan, C.E., et al., *Invisible populations: parallels between the health of people with intellectual disability and people of a refugee background.* Aust J Prim Health, 2011. **17**(3): p. 210-3.
14. Campbell, R., et al., *A Comparison of Health Access Between Permanent Residents, Undocumented Immigrants and Refugee Claimants in Toronto, Canada.* Journal of Immigrant & Minority Health, 2014. **16**(1): p. 165-176 12p.
15. Charbonneau, C.J., D.M. Kelly, and L.R. Donnelly, *Exploring the views of and challenges experienced by dental hygienists practicing in a multicultural society: A pilot study.* Canadian Journal of Dental Hygiene, 2014. **48**(4): p. 139-146 8p.
16. Cheng, I.H., A. Drillich, and P. Schattner, *Refugee experiences of general practice in countries of resettlement: a literature review.* British Journal of General Practice, 2015. **65**(632): p. e171-6 1p.
17. Cheng, I.H., et al., *Rites of passage: improving refugee access to general practice services.* Australian Family Physician, 2015. **44**(7): p. 503-507 5p.
18. Cheng, I.H., et al., *Importance of community engagement in primary health care: the case of Afghan refugees.* Australian Journal of Primary Health, 2015. **21**(3): p. 262-267 6p.
19. Clark, A., et al., *'Excuse me, do any of you ladies speak English?' Perspectives of refugee women living in South Australia: barriers to accessing primary health care and achieving the Quality Use of Medicines.* Aust J Prim Health, 2014. **20**(1): p. 92-7.
20. Cobb, T.G., *Strategies for providing cultural competent health care for Hmong Americans.* J Cult Divers, 2010. **17**(3): p. 79-83.
21. Drummond, P.D., et al., *Barriers to accessing health care services for West African refugee women living in Western Australia.* Health Care Women Int, 2011. **32**(3): p. 206-24.
22. Duguet, A.M. and B. Bévière, *Access to health care for illegal immigrants: A specific organisation in France.* European Journal of Health Law, 2011. **18**(1): p. 27-35.
23. Duke, P. and F. Brunger, *The MUN Med Gateway Project: marrying medical education and social accountability.* Can Fam Physician, 2015. **61**(2): p. e81-7.
24. Dutcher, G.A., J.C. Scott, and S.J. Arnesen, *The Refugee Health Information Network: a source of multilingual and multicultural health information.* Journal of Consumer Health on the Internet, 2008. **12**(1): p. 1-12 12p.
25. Elwell, D., et al., *Refugees in Denver and their perceptions of their health and health care.* J Health Care Poor Underserved, 2014. **25**(1): p. 128-41.
26. Farokhi, M.R., B.J. Glass, and K.M. Gureckis, *A student operated, faculty mentored dental clinic service experience at the University of Texas Health Science Center at San Antonio for the underserved refugee community: an interprofessional approach.* Tex Dent J, 2014. **131**(1): p. 27-33.
27. Fatahi, N., et al., *Experiences of Kurdish war-wounded refugees in communication with Swedish authorities through interpreter.* Patient Educ Couns, 2010. **78**(2): p. 160-5.
28. Flynn, A. and D. Flynn, *'Give us the weapon to argue': eHealth and the Somali community of Manchester.* Diversity in Health & Social Care, 2008. **5**(4): p. 255-267 13p.
29. Furber, S., et al., *A qualitative study on tobacco smoking and betel quid use among Burmese refugees in Australia.* J Immigr Minor Health, 2013. **15**(6): p. 1133-6.
30. Gele, A.A., et al., *Beyond Culture and Language: Access to Diabetes Preventive Health Services among Somali Women in Norway.* Journal of Diabetes Research, 2015. **2015**.
31. Geltman, P.L., et al., *The impact of functional health literacy and acculturation on the oral health status of somali refugees living in Massachusetts.* American Journal of Public Health, 2013. **103**(8): p. 1516-1523.
32. Geltman, P.L., et al., *Health literacy, acculturation, and the use of preventive oral health care by Somali refugees living in Massachusetts.* J Immigr Minor Health, 2014. **16**(4): p. 622-30.
33. Graham, E.A., et al., *Health services utilization by low-income limited English proficient adults.* Journal of Immigrant & Minority Health, 2008. **10**(3): p. 207-217 11p.
34. Grant, K.J., et al., *The refugee experience of acquiring a family doctor.* International Journal of Migration, Health and Social Care, 2015. **11**(1): p. 18-28.
35. Grigg-Saito, D., et al., *Building on the strengths of a Cambodian refugee community through community-based outreach.* Health Promot Pract, 2008. **9**(4): p. 415-25.
36. Grigg-Saito, D., et al., *Long-term development of a "whole community" best practice model to address health disparities in the Cambodian refugee and immigrant community of Lowell, Massachusetts.* Am J Public Health, 2010. **100**(11): p. 2026-9.
37. Gudeva Nikovska, D., et al. *Health services for migrants on the Balkan route - is Macedonia up to the challenge?* in *6th European Conference on Migrant and Ethnic Minority Health*. 2016. Oslo, Norway: https://www.duo.uio.no/bitstream/handle/10852/55205/abstract-book-eupha2016.pdf?sequence=3&isAllowed=y.
38. Hackett, J., et al., *Evaluation of three population health capacity building projects delivered by videoconferencing in NSW.* N S W Public Health Bull, 2009. **20**(11-12): p. 182-6.
39. Hadgkiss, E.J. and A.M.N. Renzaho, *The physical health status, service utilisation and barriers to accessing care for asylum seekers residing in the community: a systematic review of the literature.* Australian Health Review, 2014. **38**(2): p. 142-159 18p.
40. Haley, H.L., et al., *Primary prevention for resettled refugees from Burma: where to begin?* J Community Health, 2014. **39**(1): p. 1-10.
41. Harstad, I., et al., *Screening and treatment of latent tuberculosis in a cohort of asylum seekers in Norway.* Scand J Public Health, 2010. **38**(3): p. 275-82.
42. Hauck, F.R., et al., *Factors Influencing the Acculturation of Burmese, Bhutanese, and Iraqi Refugees Into American Society: Cross-Cultural Comparisons.* Journal of Immigrant and Refugee Studies, 2014. **12**(3): p. 331-352.
43. Helweg-Larsen, M. and L.M. Stancioff, *Acculturation matters: risk perceptions of smoking among Bosnian refugees living in the United States.* J Immigr Minor Health, 2008. **10**(5): p. 423-8.
44. Hill, L., et al., *Inter-professional learning to prepare medical and social work students for practice with refugees and asylum seekers.* Social Work Education, 2009. **28**(3): p. 298-308.
45. Hudelson, P., M. Dominice Dao, and S. Durieux-Paillard, *Quality in practice: integrating routine collection of patient language data into hospital practice.* Int J Qual Health Care, 2013. **25**(4): p. 437-42.
46. Im, H. and R. Rosenberg, *Building Social Capital Through a Peer-Led Community Health Workshop: A Pilot with the Bhutanese Refugee Community.* Journal of Community Health, 2016. **41**(3): p. 509-517.
47. Ingram, J., *The health needs of the Somali community in Bristol.* Community Pract, 2009. **82**(12): p. 26-9.
48. International Organization for Migration, *International Migration, Health and Human Rights*. 2013, IOM; WHO; UNOHCHR: Geneva, Switzerland: http://www.ohchr.org/Documents/Issues/Migration/WHO_IOM_UNOHCHRPublication.pdf.
49. International Organization for Migration, *Assessment report: the health situation at EU southern borders - migrant health, occupational health, and public health - Bulgaria - Field work 2014-2015.* 2015, IOM: https://publications.iom.int/system/files/pdf/sar_bulgaria.pdf.
50. International Organization for Migration, *Assessment report: the health situation at EU southern borders - migrant health, occupational health, and public health - Croatia - Field work 7-14 April 2014.* 2015: https://publications.iom.int/system/files/pdf/sar_croatia.pdf.
51. International Organization for Migration, *Assessment report: the health situation at EU southern borders - migrant health, occupational health, and public health - Greece - Field work 6-15 November 2013.* 2015: https://publications.iom.int/system/files/pdf/sar_greece.pdf.
52. International Organization for Migration, *Assessment report: the health situation at EU southern borders - migrant health, occupational health, and public health - Italy - Field work 4-18 September 2013.* 2015: https://publications.iom.int/system/files/pdf/sar_italy.pdf.
53. International Organization for Migration, *Assessment report: the health situation at EU southern borders - migrant health, occupational health, and public health - Malta - Field work 11-15 November 2013.* 2015: https://publications.iom.int/system/files/pdf/sar_malta.pdf.
54. International Organization for Migration, *Assessment report: the health situation at EU southern borders - migrant health, occupational health, and public health - Spain - Field work 18-27 November 2013.* 2015: https://publications.iom.int/system/files/pdf/sar_spain.pdf.
55. Ioannidi, E. *First reception of refugees entering through the Aegean. The current situation in Greek islands.* in *Public health and human rights: ensuring access to health care for refugees crossing the Mediterranean Sea*. 2015. Milano, Italy.
56. Joels, C., *Impact of national policy on the health of people seeking asylum.* Nurs Stand, 2008. **22**(31): p. 35-40.
57. Johnston, V., *Australian asylum policies: have they violated the right to health of asylum seekers?* Aust N Z J Public Health, 2009. **33**(1): p. 40-6.
58. Jones, C. and A.E. Williamson, *Volunteers working to support migrants in Glasgow: A qualitative study.* International Journal of Migration, Health and Social Care, 2014. **10**(4): p. 193-206.
59. Jonzon, R., P. Lindkvist, and E. Johansson, *A state of limbo--in transition between two contexts: Health assessments upon arrival in Sweden as perceived by former Eritrean asylum seekers.* Scand J Public Health, 2015. **43**(5): p. 548-58.
60. Joshi, C., et al., *A narrative synthesis of the impact of primary health care delivery models for refugees in resettlement countries on access, quality and coordination.* International Journal for Equity in Health, 2013. **12**(1).
61. Kaluski, D.N., et al., *Health insurance and accessibility to health services among Roma in settlements in Belgrade, Serbia - The journey from data to policy making.* Health Policy and Planning, 2015. **30**(8): p. 976-984.
62. Kandasamy, T., et al., *Obstetric risks and outcomes of refugee women at a single centre in Toronto.* J Obstet Gynaecol Can, 2014. **36**(4): p. 296-302.
63. Kay, M., C. Jackson, and C. Nicholson, *Refugee health: a new model for delivering primary health care.* Aust J Prim Health, 2010. **16**(1): p. 98-103.
64. Kay, M., et al., *Understanding quality use of medicines in refugee communities in Australian primary care: a qualitative study.* British Journal of General Practice, 2016. **66**(647): p. e397-e409 13p.
65. Klinkenberg, E., et al., *Migrant tuberculosis screening in the EU/EEA: yield, coverage and limitations.* Eur Respir J, 2009. **34**(5): p. 1180-9.
66. Kouli, E., et al., *The institutional framework regarding the rights of immigrants for access to health services in the European union.* Nursing Care and Research, 2014(36): p. 137.
67. Kreps, G.L.S., L., *Meeting the health literacy needs of immigrant’s populations.* Patient Education and Counselling, 2008. **71**: p. 328-332.
68. Lee, S.K., C.M.R. Sulaiman-Hill, and S.C. Thompson, *Providing health information for culturally and linguistically diverse women: priorities and preferences of new migrants and refugees.* Health Promotion Journal of Australia, 2013. **24**(2): p. 98-103 6p.
69. Ludwig, B. and H. Reed, *When you are here, you have high blood pressure": Liberian refugees' health and access to healthcare in Staten Island, NY.* International Journal of Migration, Health and Social Care, 2016. **12**(1): p. 26-37.
70. MacFarlane, A., et al., *Arranging and negotiating the use of informal interpreters in general practice consultations: experiences of refugees and asylum seekers in the west of Ireland.* Soc Sci Med, 2009. **69**(2): p. 210-4.
71. MacFarlane, A., et al., *Responses to language barriers in consultations with refugees and asylum seekers: a telephone survey of Irish general practitioners.* BMC Fam Pract, 2008. **9**: p. 68.
72. Manchikanti, P., *The experiences of access to primary care by afghani refugees in south east melbourne: A reflection on the public health needs of ethnic minorities.* Internal Medicine Journal 2013. **43**(S3): p. 18.
73. Mancuso, L., *Overcoming health literacy barriers: a model for action.* J Cult Divers, 2011. **18**(2): p. 60-5.
74. Matthews, A., et al. *Migration and the Media: the effect on healthcare access for asylum seekers and refugees*. in *6th European Conference on Migrant and Ethnic Minority Health*. 2016. Oslo, Norway: https://www.duo.uio.no/bitstream/handle/10852/55205/abstract-book-eupha2016.pdf?sequence=3&isAllowed=y.
75. Mayhew, M., et al., *Facilitating refugees' access to family doctors.* International Journal of Migration, Health and Social Care, 2015. **11**(1): p. 1-17.
76. McDonald, B., et al., *Refugee resettlement in regional and rural Victoria: impacts and policy issues.* Melbourne: Victorian Health Promotion Foundation, 2008.
77. McKeary, M. and B. Newbold, *Barriers to care: The challenges for Canadian refugees and their health care providers.* Journal of Refugee Studies, 2010. **23**(4): p. 523-545.
78. McMurray, J., et al., *Integrated primary care improves access to healthcare for newly arrived refugees in Canada.* J Immigr Minor Health, 2014. **16**(4): p. 576-85.
79. Médecins Sans Frontières, *NOT CRIMINALS” Médecins Sans Frontières exposes conditions for undocumented migrants and asylum seekers in Maltese detention centres*. 2009.
80. Milosevic, D., I.H. Cheng, and M.M. Smith, *The NSW refugee health service: Improving refugee access to primary care.* Australian Family Physician, 2012. **41**(3): p. 147-149.
81. Mirza, M. and A.W. Heinemann, *Service needs and service gaps among refugees with disabilities resettled in the United States.* Disabil Rehabil, 2012. **34**(7): p. 542-52.
82. Mirza, M., et al., *Barriers to Healthcare Access Among Refugees with Disabilities and Chronic Health Conditions Resettled in the US Midwest.* Journal of Immigrant & Minority Health, 2014. **16**(4): p. 733-742 10p.
83. Mitschke, D., et al., *Uncovering Health and Wellness Needs of Recently Resettled Karen Refugees from Burma.* Journal of Human Behavior in the Social Environment, 2011. **4**(21).
84. Morris, M.D., et al., *Healthcare barriers of refugee’s post-resettlement.* J Community Health, 2009. **34**(6): p. 529-38.
85. Museru, O.I., et al., *Hepatitis B virus infection among refugees resettled in the U.S.: high prevalence and challenges in access to health care.* J Immigr Minor Health, 2010. **12**(6): p. 823-7.
86. Newbold, K.B., J. Cho, and M. McKeary, *Access to Health Care: The Experiences of Refugee and Refugee Claimant Women in Hamilton, Ontario.* Journal of Immigrant and Refugee Studies, 2013. **11**(4): p. 431-449.
87. Njeru, J.W., et al., *Stories for change: development of a diabetes digital storytelling intervention for refugees and immigrants to Minnesota using qualitative methods.* BMC Public Health, 2015. **15**: p. 1311.
88. Nkulu Kalengayi, F.K. *Perspectives of asylum seekers and refugees on health assessment: “It is a requirement that benefits everyone"*. in *5th EUPHA European Conference on Migrant and Ethnic Minority Health*. 2014. Granada, Spain.
89. Norredam, M., *Migrants' access to healthcare.* Danish medical bulletin, 2011. **58**(10).
90. Norredam, M. *Migration and health: Organising access to EU health care systems for migrants*. 2016. Brussels, Belgium.
91. O'Donnell, C.A., et al., *Asylum seekers' expectations of and trust in general practice: a qualitative study.* Br J Gen Pract, 2008. **58**(557): p. e1-11.
92. O'Mara, B., *Social media, digital video and health promotion in a culturally and linguistically diverse Australia.* Health Promot Int, 2013. **28**(3): p. 466-76.
93. O'Reilly-de Brún, M., et al., *Involving migrants in the development of guidelines for communication in cross-cultural general practice consultations: a participatory learning and action research project.* BMJ Open, 2015. **5**(9).
94. Oktem, P., A.E. Akalin, and A. Gelgec Bakacak. *Migrant women's access to healthcare in Turkey*. in *6th European Conference on Migrant and Ethnic Minority Health* 2016. Oslo, Norway.
95. Okunseri, C., et al., *Hmong adults self-rated oral health: a pilot study.* Journal of Immigrant & Minority Health, 2008. **10**(1): p. 81-88 8p.
96. Pieper, H.O., P. Clerkin, and A. MacFarlane, *The impact of direct provision accommodation for asylum seekers on organisation and delivery of local primary care and social care services: A case study.* BMC Family Practice, 2011. **12**.
97. Pottie, K., et al., *Improving delivery of primary care for vulnerable migrants: Delphi consensus to prioritize innovative practice strategies.* Can Fam Physician, 2014. **60**(1): p. e32-40.
98. Power, D. and R. Pratt, *Karen refugees from Burma: focus group analysis.* International Journal of Migration, Health and Social Care, 2012. **8**(4): p. 156-166.
99. Qayyum, M.A., et al., *The provision and sharing of information between service providers and settling refugees.* Information Research, 2014. **19**(2).
100. Razavi, M.F., et al., *Experiences of the Swedish healthcare system: An interview study with refugees in need of long-term health care.* Scandinavian Journal of Public Health, 2011. **39**(3): p. 319-325.
101. Reavy, K., et al., *A new clinic model for refugee health care: adaptation of cultural safety.* Rural Remote Health, 2012. **12**: p. 1826.
102. Rechel, B., et al. *Health system responses to the influx of refugees in Europe*. in *6th European Conference on Migrant and Ethnic Minority Health*. 2016. Oslo, Norway. https://www.duo.uio.no/bitstream/handle/10852/55205/abstract-book-eupha2016.pdf?sequence=3&isAllowed=y.
103. Reichlin, R., et al. *Applying a Community-Based Participatory Research Approach to Improve Asylum-Seekers’ Access to Healthcare in Israel*. in *6th European Conference on Migrant and Ethnic Minority Health*. 2016. Oslo, Norway: https://www.duo.uio.no/bitstream/handle/10852/55205/abstract-book-eupha2016.pdf?sequence=3&isAllowed=y.
104. Rew, K.T., et al., *Immigrant and refugee health: cross-cultural communication.* FP Essent, 2014. **423**: p. 30-9.
105. Robinson, K., *Supervision Found Wanting: Experiences of Health and Social Workers in Non-Government Organisations Working with Refugees and Asylum Seekers.* Practice (09503153), 2013. **25**(2): p. 87-103 17p.
106. Ross, L., et al., *Improving the management and care of refugees in Australian hospitals: a descriptive study.* Aust Health Rev, 2016.
107. Sandliki, B., et al. *Role of NGOs in addressing the needs of Syrian refugees living in Istanbul*. in *6th European Conference on Migrant and Ethnic Minority Health*. 2016. Oslo, Norway: https://www.duo.uio.no/bitstream/handle/10852/55205/abstract-book-eupha2016.pdf?sequence=3&isAllowed=y.
108. Schulz, T.R., et al., *Improvements in patient care: videoconferencing to improve access to interpreters during clinical consultations for refugee and immigrant patients.* Aust Health Rev, 2015. **39**(4): p. 395-9.
109. Schulz, T.R., et al., *Telehealth: experience of the first 120 consultations delivered from a new refugee telehealth clinic.* Intern Med J, 2014. **44**(10): p. 981-5.
110. Scott, P., *Black African asylum seekers' experiences of health care access in an eastern German state.* International Journal of Migration, Health and Social Care, 2014. **10**(3): p. 134-147.
111. Seery, T., H. Boswell, and A. Lara, *Caring for refugee children.* Pediatrics in Review, 2015. **36**(8): p. 323-338.
112. Segala, D., et al. *Health education and HIV test offer in a population of refugees and asylum seekers: an experience in Ferrara area.* in *6th European Conference on Migrant and Ethnic Minority Health*. 2016. Oslo, Norway: https://www.duo.uio.no/bitstream/handle/10852/55205/abstract-book-eupha2016.pdf?sequence=3&isAllowed=y.
113. Sethi, B., *Service delivery on rusty health care wheels: implications for visible minority women.* J Evid Based Soc Work, 2013. **10**(5): p. 522-32.
114. Sheikh, M., et al., *Equity and access: understanding emergency health service use by newly arrived refugees.* Med J Aust, 2011. **195**(2): p. 74-6.
115. Show, J.S., et al., *The role of culture in health literacy and chronic disease screening and management.* Journal Minority Health, 2009. **11**: p. 460-467.
116. Simonnot, N., P. Chauvin, and C. Vuillermoz. *Health and access to care for migrants facing multiple vulnerabilities in Europe*. in *6th European Conference on Migrant and Ethnic Minority Health*. 2016. Oslo, Norway: https://www.duo.uio.no/bitstream/handle/10852/55205/abstract-book-eupha2016.pdf?sequence=3&isAllowed=y.
117. Spike, E.A., M.M. Smith, and M.F. Harris, *Access to primary health care services by community-based asylum seekers.* Med J Aust, 2011. **195**(4): p. 188-91.
118. Sullivan, C.H., *Partnering with community agencies to provide nursing students with cultural awareness experiences and refugee health promotion access.* J Nurs Educ, 2009. **48**(9): p. 519-22.
119. Swe, H.M. and M.W. Ross, *Refugees from Myanmar and their health care needs in the US: A qualitative study at a refugee resettlement agency.* International Journal of Migration, Health and Social Care, 2010. **6**(1): p. 15-25.
120. Szajna, A. and J. Ward, *Access to health care by refugees: a dimensional analysis.* Nurs Forum, 2015. **50**(2): p. 83-9.
121. Tastsoglou, E., et al., *(En) gendering vulnerability: Immigrant service providers' perceptions of needs, policies, and practices related to gender and women refugee claimants in Atlantic Canada.* Refuge, 2014. **30**(2): p. 67-78.
122. Taylor, K., *Asylum seekers, refugees, and the politics of access to health care: a UK perspective.* British Journal of General Practice, 2009. **59**(567): p. 765-772 8p.
123. Torres, S., et al., *Improving health equity: The promising role of community health workers in Canada.* Healthcare Policy, 2014. **10**(1): p. 73-85.
124. Torun, P., et al. *A health and health care needs assessment for the Syrian community living in Zeytinburnu district of Istanbul*. in *6th European Conference on Migrant and Ethnic Minority Health*. 2016. Oslo, Norway: https://www.duo.uio.no/bitstream/handle/10852/55205/abstract-book-eupha2016.pdf?sequence=3&isAllowed=y.
125. United Nations High Commissioner for Refugees, *Ensuring Access to Health Care: Operational Guidance on Refugee Protection and Solutions in Urban Areas.* 2011.
126. United Nations High Commissioner for Refugees, *REGIONAL REFUGEE AND MIGRANT RESPONSE PLAN FOR EUROPE. EASTERN MEDITERRANEAN AND WESTERN BALKANS ROUTE*. 2016.
127. Wagner, J., et al., *Diabetes among refugee populations: what newly arriving refugees can learn from resettled Cambodians.* Curr Diab Rep, 2015. **15**(8): p. 56.
128. Wangdahl, J., et al., *Health literacy and refugees' experiences of the health examination for asylum seekers - a Swedish cross-sectional study.* BMC Public Health, 2015. **15**: p. 1162.
129. Woodland, L., et al., *Evaluation of a school screening programme for young people from refugee backgrounds.* J Paediatr Child Health, 2016. **52**(1): p. 72-9.
130. Xiao, L.D., L. Habel, and A. De Bellis, *Perceived Challenges in Dementia Care by Vietnamese Family Caregivers and Care Workers in South Australia.* Journal of Cross-Cultural Gerontology, 2015. **30**(3): p. 333-352 20p.
131. Yun, K., et al., *Help-Seeking Behavior and Health Care Navigation by Bhutanese Refugees.* Journal of Community Health, 2016. **41**(3): p. 526-534.

**Included studies on barriers and solutions related to accessing specific healthcare services for refugees and migrants**

**Mental health services:**

1. Ahmad, F., et al., *A pilot with computer-assisted psychosocial risk-assessment for refugees.* BMC Med Inform Decis Mak, 2012. **12**: p. 71.
2. Ahmed, A., et al., *Experiences of immigrant new mothers with symptoms of depression.* Arch Womens Ment Health, 2008. **11**(4): p. 295-303.
3. Al-Obaidi, A., et al., *Incorporating Preliminary Mental Health Assessment in the Initial Healthcare for Refugees in New Jersey.* Community Ment Health J, 2015. **51**(5): p. 567-74.
4. Baarnhielm, S., et al., *Approaching the vulnerability of refugees: evaluation of cross-cultural psychiatric training of staff in mental health care and refugee reception in Sweden.* BMC Med Educ, 2014. **14**: p. 207.
5. Baarnhielm, S., C. Javo, and M.O. Mosko, *Opening up mental health service delivery to cultural diversity: current situation, development and examples from three northern European countries.* Adv Psychosom Med, 2013. **33**: p. 40-55.
6. Bell, P. and E. Zech, *Access to mental health for asylum seekers in the European union: An analysis of disparities between legal rights and reality.* Archives of Public Health, 2009. **67**(1): p. 30-44.
7. Berthold, S.M., et al., *Comorbid mental and physical health and health access in Cambodian refugees in the US.* J Community Health, 2014. **39**(6): p. 1045-52.
8. Boynton, L., et al., *The role of stigma and state in the mental health of Somalis.* J Psychiatr Pract, 2010. **16**(4): p. 265-8.
9. Brandon Chen, Y.Y., et al., *Improving access to mental health services for racialized immigrants, refugees, and non- status people living with HIV/AIDS.* Journal of Health Care for the Poor and Underserved, 2015. **26**(2): p. 505-518.
10. Chiumento, A., et al., *School-based mental health service for refugee and asylum seeking children: multi-agency working, lessons for good practice.* Journal of Public Mental Health, 2011. **10**(3): p. 164-177 14p.
11. Colucci, E., et al., *In or out? Barriers and facilitators to refugee-background young people accessing mental health services.* Transcultural Psychiatry, 2015. **52**(6): p. 766-790.
12. Colucci, E., et al., *The utilisation of mental health services by children and young people from a refugee background: a systematic literature review.* International Journal of Culture and Mental Health, 2014. **7**(1): p. 86-108.
13. Derluyn, I. and E. Broekaert, *Unaccompanied refugee children and adolescents: the glaring contrast between a legal and a psychological perspective.* Int J Law Psychiatry, 2008. **31**(4): p. 319-30.
14. Ellis, B.H., et al., *New directions in refugee youth mental health services: Overcoming barriers to engagement.* Journal of Child and Adolescent Trauma, 2011. **4**(1): p. 69-85.
15. 15. Furler, J., et al., *Managing depression among ethnic communities: a qualitative study.* Ann Fam Med, 2010. **8**(3): p. 231-6.
16. 16. Ginieniewicz, J. and K. McKenzie, *Mental health of Latin Americans in Canada: a literature review.* Int J Soc Psychiatry, 2014. **60**(3): p. 263-73.
17. 17. Grazier, K.L., *Integrating behavioral health care and primary care: Application of a clinical and economic model in culturally diverse communities.* International Journal of Public Administration, 2008. **31**(14): p. 1532-1547.
18. 18. Hassan, G., et al., *Mental health and psychosocial wellbeing of Syrians affected by armed conflict.* Epidemiology and Psychiatric Sciences, 2016. **25**(2): p. 129-141.
19. 19. Henley, J. and J. Robinson, *Mental health issues among refugee children and adolescents.* Clinical Psychologist, 2011. **15**(2): p. 51-62 12p.
20. 20. Hughes, G., *Finding a voice through 'The Tree of Life': a strength-based approach to mental health for refugee children and families in schools.* Clin Child Psychol Psychiatry, 2014. **19**(1): p. 139-53.
21. 21. Jensen, N.K., et al., *Patient experienced continuity of care in the psychiatric healthcare system—a study including immigrants, refugees and ethnic Danes.* International Journal of Environmental Research and Public Health, 2014. **11**(9): p. 9739-9759.
22. 22. Jensen, N.K., et al., *How do general practitioners experience providing care to refugees with mental health problems? A qualitative study from Denmark.* BMC Fam Pract, 2013. **14**: p. 17.
23. 23. Kaczorowski, J.A., et al., *Adapting clinical services to accommodate needs of refugee populations.* Professional Psychology: Research & Practice, 2011. **42**(5): p. 361-367 7p.
24. 24. Kieft, B., et al., *Paraprofessional counselling within asylum seekers' groups in the Netherlands: transferring an approach for a non-Western context to a European setting.* Transcultural Psychiatry, 2008. **45**(1): p. 105-120 16p.
25. 25. Kirmayer, L.J., et al., *Common mental health problems in immigrants and refugees: general approach in primary care.* Cmaj, 2011. **183**(12): p. E959-67.
26. 26. Lee, H.Y., et al., *Mental health literacy in Hmong and Cambodian elderly refugees: a barrier to understanding, recognizing, and responding to depression.* Int J Aging Hum Dev, 2010. **71**(4): p. 323-44.
27. 27. Lee, S.K., S.C. Thompson, and D. Amorin-Woods, *One service, many voices: enhancing consumer participation in a primary health service for multicultural women.* Quality in Primary Care, 2009. **17**(1): p. 63-69 7p.
28. 28. Lindert, J., et al., *Mental health, health care utilisation of migrants in Europe.* European Psychiatry, 2008. **23**(SUPPL. 1): p. 14-20.
29. 29. Majumder, P., et al., *'This doctor, I not trust him, I'm not safe': the perceptions of mental health and services by unaccompanied refugee adolescents.* Int J Soc Psychiatry, 2015. **61**(2): p. 129-36.
30. 30. Maroney, P., M. Potter, and V.R. Thacore, *Experiences in occupational therapy with Afghan clients in Australia.* Aust Occup Ther J, 2014. **61**(1): p. 13-9.
31. 31. McCleary, J.S., P.J. Shannon, and T.L. Cook, *Connecting Refugees to Substance Use Treatment: A Qualitative Study.* Social Work in Public Health, 2016. **31**(1): p. 1-8 8p.
32. 32. McKenzie, K., *Issues and Options for Improving Services for Diverse Populations.* Canadian Journal of Community Mental Health, 2016. **34**(4): p. 69-88.
33. 33. Fang, M.L., et al., *Experiencing 'pathologized presence and normalized absence'; understanding health related experiences and access to health care among Iraqi and Somali asylum seekers, refugees and persons without legal status.* BMC Public Health, 2015. **15**(1): p. 1-12 12p.
34. 34. Mirdal, G.M., E. Ryding, and M. Essendrop Sondej, *Traumatized refugees, their therapists, and their interpreters: three perspectives on psychological treatment.* Psychol Psychother, 2012. **85**(4): p. 436-55.
35. 35. Mucic, D., *Transcultural telepsychiatry and its impact on patient satisfaction.* J Telemed Telecare, 2010. **16**(5): p. 237-42.
36. 36. Nazzal, K.H., et al., *An innovative community-oriented approach to prevention and early intervention with refugees in the United States.* Psychological Services, 2014. **11**(4): p. 477-485.
37. 37. Piwowarczyk, L., et al., *Congolese and somali beliefs about mental health services.* Journal of Nervous & Mental Disease, 2014. **202**(3): p. 209-216 8p.
38. 38. Platform for International Cooperation on Undocumented Migrants, *Undocumented Children in Europe: Invisible Victims of Immigration Restrictions. Daphne II Programme 2007 – 2013*. 2008.
39. 39. Posselt, M., et al., *Merging perspectives: obstacles to recovery for youth from refugee backgrounds with comorbidity.* Australas Psychiatry, 2015. **23**(3): p. 293-9.
40. 40. Priebe, S., et al., *Good practice in mental health care for socially marginalised groups in Europe: A qualitative study of expert views in 14 countries.* BMC Public Health, 2012. **12**(1).
41. 41. Rabiee, F. and P. Smith. *Equity in Mental Health Service Provision for African Caribbean, Black African Refugees and Asylum Seekers*. in *6th European Conference on Migrant and Ethnic Minority Health*. 2016. Oslo, Norway.
42. 42. Redwood-Campbell, L., et al., *Understanding the health of refugee women in host countries: lessons from the Kosovar re-settlement in Canada.* Prehosp Disaster Med, 2008. **23**(4): p. 322-7.
43. 43. Rousseau, C. and J. Guzder, *School-based prevention programs for refugee children.* Child Adolesc Psychiatr Clin N Am, 2008. **17**(3): p. 533-49, viii.
44. 44. Russo, A., et al., *A qualitative exploration of the emotional wellbeing and support needs of new mothers from Afghanistan living in Melbourne, Australia.* BMC Pregnancy Childbirth, 2015. **15**: p. 197.
45. 45. Sandhu, S., et al., *Experiences with treating immigrants: a qualitative study in mental health services across 16 European countries.* Soc Psychiatry Psychiatr Epidemiol, 2013. **48**(1): p. 105-16.
46. 46. Simich, L., *Health literacy, immigrants and mental health.* Canadian Issues / Thèmes Canadiens, 2010(Summer): p. 17-22.
47. 47. Thomson, M.S., et al., *Improving Immigrant Populations’ Access to Mental Health Services in Canada: A Review of Barriers and Recommendations.* Journal of Immigrant and Minority Health, 2015. **17**(6): p. 1895-1905.
48. 48. Weine, S., et al., *Evaluating a multiple-family group access intervention for refugees with PTSD.* J Marital Fam Ther, 2008. **34**(2): p. 149-64.
49. 49. Wohler, Y. and J.A. Dantas, *Barriers Accessing Mental Health Services Among Culturally and Linguistically Diverse (CALD) Immigrant Women in Australia: Policy Implications.* J Immigr Minor Health, 2016.
50. 50. Wollersheim, D., et al., *Constant connections: piloting a mobile phone-based peer support program for Nuer (southern Sudanese) women.* Aust J Prim Health, 2013. **19**(1): p. 7-13.

**Child and adolescent health services:**

1. Abbing, H.D., *Age determination of unaccompanied asylum seeking minors in the European Union: a health law perspective.* Eur J Health Law, 2011. **18**(1): p. 11-25.
2. Alayarian, A., *Children, torture and psychological consequences.* Torture, 2009. **19**(2): p. 145-56.
3. Anders, A.D.P. and J.N. Lester, *Navigating authoritarian power in the United States: Families with refugee status and allegorical representation.* Cultural Studies - Critical Methodologies, 2015. **15**(3): p. 169-179.
4. Burchill, J., *Safeguarding vulnerable families: work with refugees and asylum seekers.* Community Pract, 2011. **84**(2): p. 23-6.
5. Chauvin, P., et al., *Access to healthcare for people facing multiple vulnerabilities in health.* 2015, Doctors of the World - Médecins du monde International Network: Paris, France.
6. Chauvin, P., N. Simonnot, and C. Vuillermoz. *Non access to vaccinations among migrant and ethnic minorities’ children: analysis from Doctors of the World International Network Observatory*. in *6th European Conference on Migrant and Ethnic Minority Health*. 2016. Oslo, Norway
7. Chiumento, A., et al., *School-based mental health service for refugee and asylum seeking children: multi-agency working, lessons for good practice.* Journal of Public Mental Health, 2011. **10**(3): p. 164-177 14p.
8. Colucci, E., et al., *In or out? Barriers and facilitators to refugee-background young people accessing mental health services.* Transcultural Psychiatry, 2015. **52**(6): p. 766-790.
9. Colucci, E., et al., *The utilisation of mental health services by children and young people from a refugee background: a systematic literature review.* International Journal of Culture and Mental Health, 2014. **7**(1): p. 86-108.
10. Derluyn, I. and E. Broekaert, *Unaccompanied refugee children and adolescents: the glaring contrast between a legal and a psychological perspective.* Int J Law Psychiatry, 2008. **31**(4): p. 319-30.
11. Ellis, B.H., et al., *New directions in refugee youth mental health services: Overcoming barriers to engagement.* Journal of Child and Adolescent Trauma, 2011. **4**(1): p. 69-85.
12. Gibbs, L., et al., *An exploratory trial implementing a community-based child oral health promotion intervention for Australian families from refugee and migrant backgrounds: a protocol paper for Teeth Tales.* BMJ Open, 2014. **4**(3): p. e004260.
13. Henley, J. and J. Robinson, *Mental health issues among refugee children and adolescents.* Clinical Psychologist, 2011. **15**(2): p. 51-62 12p.
14. Hjern, A., M. Brendler-Lindqvist, and M. Norredam, *Age assessment of young asylum seekers.* Acta Paediatr, 2012. **101**(1): p. 4-7.
15. Hughes, G., *Finding a voice through 'The Tree of Life': a strength-based approach to mental health for refugee children and families in schools.* Clin Child Psychol Psychiatry, 2014. **19**(1): p. 139-53.
16. Nicol, P., et al., *Informing a culturally appropriate approach to oral health and dental care for pre-school refugee children: a community participatory study.* BMC Oral Health, 2014. **14**: p. 69.
17. Platform for International Cooperation on Undocumented Migrants, *Undocumented Children in Europe: Invisible Victims of Immigration Restrictions. Daphne II Programme 2007 – 2013*. 2008.
18. Poureslami, I., et al., *Bridging immigrants and refugees with early childhood development services: partnership research in the development of an effective service model.* Early Child Development and Care, 2013. **183**(12): p. 1924-1942.
19. Ratnam, S., P.-A. Crisinel, and U. Simeoni. *The "migrant kit": a new guide for migrant-friendly care in a Swiss paediatric hospital*. in *6th European Conference on Migrant and Ethnic Minority Health*. 2016. Oslo, Norway.
20. Riggs, E., et al., *Accessing maternal and child health services in Melbourne, Australia: reflections from refugee families and service providers.* BMC Health Serv Res, 2012. **12**: p. 117.
21. Riggs, E., et al., *'We are all scared for the baby': promoting access to dental services for refugee background women during pregnancy.* BMC Pregnancy & Childbirth, 2016. **16**: p. 1-11 11p.
22. Rousseau, C. and J. Guzder, *School-based prevention programs for refugee children.* Child Adolesc Psychiatr Clin N Am, 2008. **17**(3): p. 533-49, viii.
23. Sandahl, H., et al., *Policies of access to healthcare services for accompanied asylum-seeking children in the Nordic countries.* Scand J Public Health, 2013. **41**(6): p. 630-6.
24. Sheikh, M. and C.R. MacIntyre, *The impact of intensive health promotion to a targeted refugee population on utilisation of a new refugee paediatric clinic at the children's hospital at Westmead.* Ethn Health, 2009. **14**(4): p. 393-405.
25. United Nations High Commissioner for Refugees, *Study of the Office of the United Nations High Commissioner for Human Rights on challenges and best practices in the implementation of the international framework for the protection of the rights of the child in the context of migration (A /HRC/15/29) Geneva, 2010. Human Rights Council. Fifteenth session.*
26. Vanthuyne, K., et al., *Health workers' perceptions of access to care for children and pregnant women with precarious immigration status: health as a right or a privilege?* Soc Sci Med, 2013. **93**: p. 78-85.
27. Vermette, D., et al., *Healthcare Access for Iraqi Refugee Children in Texas: Persistent Barriers, Potential Solutions, and Policy Implications.* Journal of Immigrant & Minority Health, 2015. **17**(5): p. 1526-1536 11p.
28. Wahoush, E.O., *Equitable health-care access: the experiences of refugee and refugee claimant mothers with an ill preschooler.* Can J Nurs Res, 2009. **41**(3): p. 186-206.
29. Wollscheid, S., et al., *Effect of Interventions to Facilitate Communication Between Families or Single Young People with Minority Language Background and Public Services: A Systematic Review*. 2015.
30. Woodland, L., et al., *Health service delivery for newly arrived refugee children: a framework for good practice.* J Paediatr Child Health, 2010. **46**(10): p. 560-7.

**Victim of violence health services:**

1. Akar, F.A., et al., *The Istanbul protocol (manual on the effective investigation and documentation of torture and other cruel, inhuman or degrading treatment or punishment): implementation and education in Israel.* Isr Med Assoc J, 2014. **16**(3): p. 137-41.
2. Alayarian, A., *Children, torture and psychological consequences.* Torture, 2009. **19**(2): p. 145-56.
3. Asgary, R. and C.L. Smith, *Ethical and professional considerations providing medical evaluation and care to refugee asylum seekers.* Am J Bioeth, 2013. **13**(7): p. 3-12.
4. Borland, R. and C. Zimmerman, *CARING FOR TRAFFICKED PERSONS Training FACILITATOR’S GUIDE*. 2009.
5. Borland, R. and C. Zimmerman, *CARING FOR TRAFFICKED PERSONS guidance fir health professionals*. 2012.
6. Briones-Vozmediano, E., D. La Parra, and C. Vives-Cases, *Barriers and facilitators to effective coverage of Intimate Partner Violence services for immigrant women in Spain.* Health Expectations, 2015. **18**(6): p. 2994-3006.
7. Crosby, S.S., *Primary care management of non-English-speaking refugees who have experienced trauma: a clinical review.* Jama, 2013. **310**(5): p. 519-28.
8. Ginieniewicz, J. and K. McKenzie, *Mental health of Latin Americans in Canada: a literature review.* Int J Soc Psychiatry, 2014. **60**(3): p. 263-73.
9. Hassan, G., et al., *Mental health and psychosocial wellbeing of Syrians affected by armed conflict.* Epidemiology and Psychiatric Sciences, 2016. **25**(2): p. 129-141.
10. Keygnaert I, et al., *What is the evidence on the reduction of inequalities in accessibility and quality of maternal health care delivery for migrants? A review of the existing evidence in the WHO European Region*, in *Health Evidence Network (HEN) synthesis report*, C.W.R.O.f. Europe, Editor. 2016.

**Sexual and reproductive health services:**

1. Balaam, M.-C., et al., *A qualitative review of migrant women's perceptions of their needs and experiences related to pregnancy and childbirth.* Journal of Advanced Nursing, 2013. **69**(9): p. 1919-1930 12p.
2. Balaam, M.C., et al., *'We make them feel special': The experiences of voluntary sector workers supporting asylum seeking and refugee women during pregnancy and early motherhood.* Midwifery, 2016. **34**: p. 133-140.
3. Balachandra, S.K., et al., *Family-centered maternity care for deaf refugees: the patient-centered medical home in action.* Fam Syst Health, 2009. **27**(4): p. 362-7.
4. Bennett, S. and J. Scammell, *Midwives caring for asylum-seeking women: research findings.* Pract Midwife, 2014. **17**(1): p. 9-12.
5. Briscoe, L. and T. Lavender, *Exploring maternity care for asylum seekers and refugees.* British Journal of Midwifery, 2009. **17**(1): p. 17–23.
6. Brown, E., et al., *"They get a C-section...they gonna die": Somali women's fears of obstetrical interventions in the United States.* J Transcult Nurs, 2010. **21**(3): p. 220-7.
7. Correa-Velez, I. and J. Ryan, *Developing a best practice model of refugee maternity care.* Women Birth, 2012. **25**(1): p. 13-22.
8. Degni, F., et al., *Communication and cultural issues in providing reproductive health care to immigrant women: health care providers' experiences in meeting the needs of [corrected] Somali women living in Finland.* J Immigr Minor Health, 2012. **14**(2): p. 330-43.
9. DeStephano, C.C., P.M. Flynn, and B.C. Brost, *Somali prenatal education video use in a United States obstetric clinic: a formative evaluation of acceptability.* Patient Educ Couns, 2010. **81**(1): p. 137-41.
10. Fang, D.M. and D.L. Baker, *Barriers and facilitators of cervical cancer screening among women of Hmong origin.* J Health Care Poor Underserved, 2013. **24**(2): p. 540-55.
11. Feldman, R., *When maternity doesn’t matter Dispersing pregnant women seeking asylum*. 2013, Maternity Action & Refugee Council.
12. Gagnon, A.J., et al., *Do referrals work? Responses of childbearing newcomers to referrals for care.* J Immigr Minor Health, 2010. **12**(4): p. 559-68.
13. Goosen, S., I.E. van Oostrum, and M.L. Essink-Bot, *[Obstetric outcomes and expressed health needs of pregnant asylum seekers: a literature survey].* Ned Tijdschr Geneeskd, 2010. **154**(47): p. A2318.
14. Gurnah, K., et al., *Lost in Translation: Reproductive Health Care Experiences of Somali Bantu Women in Hartford, Connecticut.* Journal of Midwifery & Women's Health, 2011. **56**(4): p. 340-346 7p.
15. Haith-Cooper, M. and G. Bradshaw, *Meeting the health and social needs of pregnant asylum seekers, midwifery students' perspectives: part 1; dominant discourses and midwifery students.* Nurse Educ Today, 2013. **33**(9): p. 1008-13.
16. Haworth, R.J., et al., *Knowledge, attitudes, and practices for cervical cancer screening among the Bhutanese refugee community in Omaha, Nebraska.* J Community Health, 2014. **39**(5): p. 872-8.
17. Higginbottom, G.M., et al., *"I have to do what I believe": Sudanese women's beliefs and resistance to hegemonic practices at home and during experiences of maternity care in Canada.* BMC Pregnancy Childbirth, 2013. **13**: p. 51.
18. Iliadi, P., *Refugee women in Greece: - a qualitative study of their attitudes and experience in antenatal care.* Health Science Journal, 2008. **2**(3): p. 173-180 8p.
19. Keygnaert I, et al., *What is the evidence on the reduction of inequalities in accessibility and quality of maternal health care delivery for migrants? A review of the existing evidence in the WHO European Region*, in *Health Evidence Network (HEN) synthesis report*, C.W.R.O.f. Europe, Editor. 2016.
20. Keygnaert, I., et al., *Sexual health is dead in my body: participatory assessment of sexual health determinants by refugees, asylum seekers and undocumented migrants in Belgium and The Netherlands.* BMC Public Health, 2014. **14**: p. 416.
21. Kowal, S.P., C.G. Jardine, and T.M. Bubela, *"If they tell me to get it, I'll get it. If they don't...": Immunization decision-making processes of immigrant mothers.* Can J Public Health, 2015. **106**(4): p. e230-5.
22. Kurth, E., et al., *Reproductive health care for asylum-seeking women - a challenge for health professionals.* BMC Public Health, 2010. **10**: p. 659.
23. Matthews, A., et al. *How do asylum seeking and refugee women perceive and respond to preventive health care? Cervical Screening as a case study.* in *6th European Conference on Migrant and Ethnic Minority Health*. 2016. Oslo, Norway.
24. McMichael, C. and S. Gifford, *"It is Good to Know Now...Before it's Too Late": Promoting sexual health literacy amongst resettled young people with refugee backgrounds.* Sexuality and Culture, 2009. **13**(4): p. 218-236.
25. Merry, L.A., et al., *Refugee claimant women and barriers to health and social services post-birth.* Can J Public Health, 2011. **102**(4): p. 286-90.
26. Murray, L., et al., *The experiences of African women giving birth in Brisbane, Australia.* Health Care Women Int, 2010. **31**(5): p. 458-72.
27. O'Mahony, J. and T. Donnelly, *Immigrant and refugee women's post-partum depression help-seeking experiences and access to care: a review and analysis of the literature.* J Psychiatr Ment Health Nurs, 2010. **17**(10): p. 917-28.
28. O'Mahony, J.M. and T.T. Donnelly, *How does gender influence immigrant and refugee women's postpartum depression help-seeking experiences?* J Psychiatr Ment Health Nurs, 2013. **20**(8): p. 714-25.
29. Odunukan, O.W., et al., *Provider and interpreter preferences among Somali women in a primary care setting.* J Prim Care Community Health, 2015. **6**(2): p. 105-10.
30. Percac-Lima, S., et al., *Decreasing disparities in breast cancer screening in refugee women using culturally tailored patient navigation.* J Gen Intern Med, 2013. **28**(11): p. 1463-8.
31. Percac-Lima, S., et al., *Patient navigation to improve breast cancer screening in Bosnian refugees and immigrants.* J Immigr Minor Health, 2012. **14**(4): p. 727-30.
32. Pimentel, V.M. and M.J. Eckardt, *More than interpreters needed: the specialized care of the immigrant pregnant patient.* Obstet Gynecol Surv, 2014. **69**(8): p. 490-500.
33. Redwood-Campbell, L., et al., *Understanding the health of refugee women in host countries: lessons from the Kosovar re-settlement in Canada.* Prehosp Disaster Med, 2008. **23**(4): p. 322-7.
34. Reynolds, B. and J. White, *Seeking asylum and motherhood: health and wellbeing needs.* Community Pract, 2010. **83**(3): p. 20-3.
35. Riggs, E., et al., *Accessing maternal and child health services in Melbourne, Australia: reflections from refugee families and service providers.* BMC Health Serv Res, 2012. **12**: p. 117.
36. Riggs, E., et al., *'We are all scared for the baby': promoting access to dental services for refugee background women during pregnancy.* BMC Pregnancy & Childbirth, 2016. **16**: p. 1-11 11p.
37. Saadi, A., B. Bond, and S. Percac-Lima, *Perspectives on preventive health care and barriers to breast cancer screening among Iraqi women refugees.* J Immigr Minor Health, 2012. **14**(4): p. 633-9.
38. Sinha, S., S. Uppal, and A. Pryce, *'I had to cry': exploring sexual health with young separated asylum seekers in East London.* Diversity in Health & Social Care, 2008. **5**(2): p. 101-111 11p.
39. Sudbury, H. and A. Robinson, *Barriers to sexual and reproductive health care for refugee and asylum-seeking women.* British Journal of Midwifery, 2016. **24**(4): p. 275-281.
40. Tobin, C., J. Murphy-Lawless, and C.T. Beck, *Childbirth in exile: asylum seeking women's experience of childbirth in Ireland.* Midwifery, 2014. **30**(7): p. 831-8.
41. Ussher, J.M., et al., *Purity, Privacy and Procreation: Constructions and Experiences of Sexual and Reproductive Health in Assyrian and Karen Women Living in Australia.* Sexuality and Culture, 2012. **16**(4): p. 467-485.
42. Vanthuyne, K., et al., *Health workers' perceptions of access to care for children and pregnant women with precarious immigration status: health as a right or a privilege?* Soc Sci Med, 2013. **93**: p. 78-85.
43. Wojnar, D.M., *Perinatal Experiences of Somali Couples in the United States.* JOGNN: Journal of Obstetric, Gynecologic & Neonatal Nursing, 2015. **44**(3): p. 358-369 12p.
44. Yelland, J., et al., *Maternity services are not meeting the needs of immigrant women of non-English speaking background: Results of two consecutive Australian population based studies.* Midwifery, 2015. **31**(7): p. 664-670.
45. Yelland, J., et al., *Compromised communication: A qualitative study exploring Afghan families and health professionals' experience of interpreting support in Australian maternity care.* BMJ Quality and Safety, 2016. **25**(4): p. e1.
